# Phase Separation Mediates NUP98 Fusion Oncoprotein Leukemic Transformation

**DOI:** 10.1158/2159-8290.CD-21-0674

**Published:** 2021-12-13

**Authors:** Bappaditya Chandra, Nicole L. Michmerhuizen, Hazheen K. Shirnekhi, Swarnendu Tripathi, Brittany J. Pioso, David W. Baggett, Diana M. Mitrea, Ilaria Iacobucci, Michael R. White, Jingjing Chen, Cheon-Gil Park, Huiyun Wu, Stanley Pounds, Anna Medyukhina, Khaled Khairy, Qingsong Gao, Chunxu Qu, Sherif Abdelhamed, Scott D. Gorman, Simranjot Bawa, Carolyn Maslanka, Swati Kinger, Priyanka Dogra, Mylene C. Ferrolino, Danika Di Giacomo, Cristina Mecucci, Jeffery M. Klco, Charles G. Mullighan, Richard W. Kriwacki

**Affiliations:** 1 Department of Structural Biology, St. Jude Children's Research Hospital, Memphis, Tennessee.; 2 Department of Pathology, St. Jude Children's Research Hospital, Memphis, Tennessee.; 3 Integrated Biomedical Sciences Program, the University of Tennessee Health Science Center, Memphis, Tennessee.; 4 Department of Hematology, St. Jude Children's Research Hospital, Memphis, Tennessee.; 5 Department of Biostatistics, St. Jude Children's Research Hospital, Memphis, Tennessee.; 6 Center for Bioimage Informatics, St. Jude Children's Research Hospital Memphis, Tennessee.; 7 Rhodes College, Memphis, Tennessee.; 8 Department of Medicine and Surgery, University of Perugia, Perugia, Italy.; 9 Department of Microbiology, Immunology and Biochemistry, University of Tennessee Health Sciences Center, Memphis, Tennessee.

## Abstract

NUP98 fusion oncoproteins (FO) are drivers in pediatric leukemias and many transform hematopoietic cells. Most NUP98 FOs harbor an intrinsically disordered region from NUP98 that is prone to liquid–liquid phase separation (LLPS) *in vitro*. A predominant class of NUP98 FOs, including NUP98–HOXA9 (NHA9), retains a DNA-binding homeodomain, whereas others harbor other types of DNA- or chromatin-binding domains. NUP98 FOs have long been known to form puncta, but long-standing questions are how nuclear puncta form and how they drive leukemogenesis. Here we studied NHA9 condensates and show that homotypic interactions and different types of heterotypic interactions are required to form nuclear puncta, which are associated with aberrant transcriptional activity and transformation of hematopoietic stem and progenitor cells. We also show that three additional leukemia-associated NUP98 FOs (NUP98–PRRX1, NUP98–KDM5A, and NUP98–LNP1) form nuclear puncta and transform hematopoietic cells. These findings indicate that LLPS is critical for leukemogenesis by NUP98 FOs.

**Significance:**

We show that homotypic and heterotypic mechanisms of LLPS control NUP98–HOXA9 puncta formation, modulating transcriptional activity and transforming hematopoietic cells. Importantly, these mechanisms are generalizable to other NUP98 FOs that share similar domain structures. These findings address long-standing questions on how nuclear puncta form and their link to leukemogenesis.

*This article is highlighted in the In This Issue feature, p. 873*

## Teaser

NUP98 fusion oncoproteins promote leukemogenesis by undergoing phase separation in the nuclei of hematopoietic cells to form hundreds of punctate transcriptional condensates that drive aberrant gene expression and induce myeloid transformation.

## Introduction

Chromosomal translocations involving the nucleoporin 98 (*NUP98*) gene are observed in various hematologic malignancies and are a hallmark of high-risk childhood leukemias. NUP98 fusion oncoproteins (FO) occur in approximately 5% of all pediatric patients with acute myeloid leukemia (AML; refs. 1–5). *NUP98* rearrangement shows increased prevalence within specific AML subtypes, including those with monocytic, megakaryoblastic, and erythroid differentiation (3, 6–9). Moreover, 50% of children with chemotherapy-resistant AML bear NUP98 FOs (10), and there are currently no effective targeted therapeutic strategies for these patients. Thus, understanding the molecular mechanism by which NUP98 FOs drive cancer is needed to offer pediatric patients with AML opportunities for improved clinical outcomes in the future.

In normal cells, NUP98 primarily functions within the nuclear pore complex (NPC), with additional roles in transcription and mitosis (11–15). The N-terminal portion of the NUP98 protein is an intrinsically disordered region (IDR) enriched in phenylalanine-glycine (FG) motifs (16) interrupted by a Gle2-binding-sequence (GLEBS) domain (17). NUP98 FOs universally include the N-terminal region of NUP98 linked to C-terminal regions from one of >30 partner genes, almost all of which exhibit one or more DNA- or chromatin-binding domains (ref. 18; Supplementary Table S1). The most prevalent type of fusion partners exhibit a DNA-binding homeodomain (18), with NUP98–HOXA9 the first identified and most well studied (19, 20). Also prevalent are NUP98 fusion partners that are epigenetic regulators, many of which exhibit chromatin-binding PHD and/or SET domains, including NUP98–KDM5A (18). A distinguishing but often overlooked feature of NUP98 FOs is that they form nuclear foci or puncta (Supplementary Table S1). Although puncta formed by NUP98 FOs have been associated with leukemogenic phenotypes in hematopoietic cells (21), how puncta form and how they are linked with altered cellular behavior are poorly understood.

Importantly, data from multiple sources indicate influences of the NUP98 FOs on chromatin state, including accessibility for gene expression. For example, NUP98–HOXA9 (NHA9), NUP98–NSD1, NUP98–KDM5A, and NUP98–PHF23 bind at the *HoxA* gene cluster, a locus that is otherwise highly compacted and repressed during differentiation (21–24). Results from model systems and patient samples bearing NUP98 FOs, including NHA9 and others (6, 7, 25, 26), show that chromatin remodeling drives aberrant transcription and upregulation of *HOX* genes. In addition, transcriptional cofactors (27), including CREBBP (28), EP300 (28), XPO1 (CRM1; ref. 29), and KMT2A (23, 30) or WDR–SET1–COMPASS complexes (23, 30), interact with NUP98's IDR and mediate the transcriptional signature unique to *NUP98*-rearranged hematologic malignancies (22). However, how chromatin remodeling and aberrant transcriptional regulation are orchestrated within the nuclear puncta formed by NUP98 FOs is only beginning to be understood (31).

The transcriptional machinery is organized within submicron-size puncta termed transcriptional condensates (32) that form through liquid–liquid phase separation (LLPS; refs. 33–37). Many transcriptional regulators, including transcription factors, coregulators, and RNA polymerase II, contain IDRs that promote multivalent interactions that drive LLPS *in vitro* and in cells (38). Biomolecules within transcriptional condensates are highly dynamic, promoting biochemical processes within them (36). Transcription factors often display a disordered, LLPS-prone activation domain linked to a sequence-specific DNA-binding domain and promote the formation of transcriptional condensates at regulatory DNA sites within genes (34). The LLPS-prone IDRs of transcriptional coregulators and RNA polymerase II enable their copartitioning with transcription factors within condensates (34).

**Figure 1.**
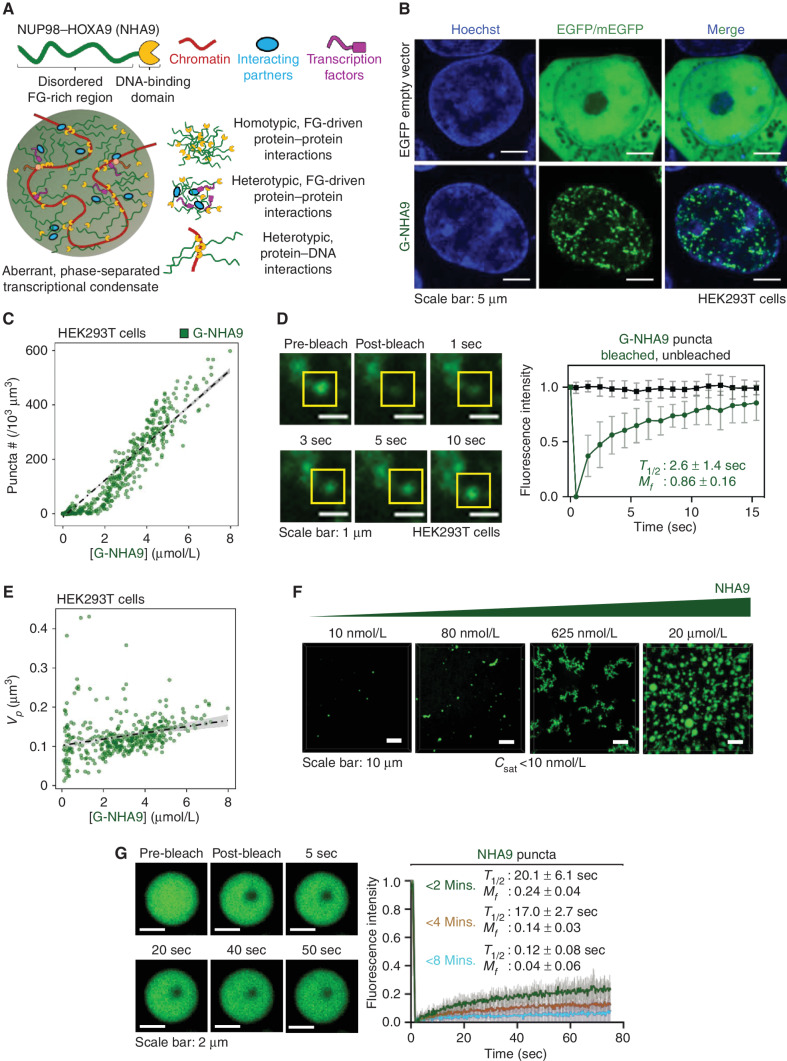
LLPS by the N-terminal FG motif–rich IDR governs the puncta-forming behavior of G-NHA9 in cells and *in vitro*. **A,** Scheme depicting the hypothesized role of both homotypic and heterotypic interactions in the formation of aberrant transcriptional condensates by NHA9 via LLPS. **B,** Representative confocal microscopy images of live HEK293T cells expressing EGFP empty vector (top, green) or G-NHA9 (bottom, green). DNA is stained with Hoechst dye (blue). **C,** The number of puncta per 10^3^ μm^3^ nuclear volume (puncta #,/10^3^ μm^3^) versus the total nuclear concentration of the G-NHA9 construct [G-NHA9]. The dotted lines represent linear fitting for visualization purposes, and the gray area indicates 95% confidence interval. Number of cells (*n*) = 935, including the cells with zero punctum. **D,** Confocal micrographs of fluorescence recovery (inside yellow box) of a single G-NHA9 punctum in HEK293T cells at different times after photobleaching (FRAP, left). FRAP recovery curve for a photobleached punctum (green, right) and an unbleached punctum (black, right). Individual puncta were manually tracked at different times, and recovery was plotted as the mean ± SD (*n* = 20). **E,** The average puncta volume (*V_p_*, μm^3^) versus the total nuclear concentration of the G-NHA9 construct [G-NHA9]. The dotted lines represent linear fitting for visualization purposes, and the gray area indicates 95% confidence interval. Number of cells (*n*) = 378, excluding the cells with zero punctum. **F,** Confocal fluorescence micrographs of Alexa 488–labeled NHA9 condensates prepared *in vitro* with increasing protein concentration. The micrographs are presented as maximum intensity projections of 13 confocal planes offset by 0.5 μm per plane. Saturation concentration (*C*_sat_) is less than 10 nmol/L. **G,** Confocal micrographs of fluorescence recovery of 20 μmol/L NHA9 (mixed with 200 nmol/L Alexa 488–labeled NHA9) within condensates at multiple time points acquired within 2 minutes of initiation of phase separation (left). FRAP recovery curves for NHA9 condensates within 2 (green), 4 (brown), and 8 (cyan) minutes of formation (right). Data are plotted as mean ± SD (*n* = 15). *T*_1/2_ represents the half-life of the fluorescence recovery, and *M_f_* represents the mobile fraction.

We and others (27, 31) observed similarities between the domain structure of transcription factors and NUP98 FOs, specifically, the presence of a phase separation–prone IDR and DNA- or chromatin-binding domains. The N-terminal FG-rich IDR of NUP98 (NUP98-N), common to all leukemia-associated NUP98 FOs (18), undergoes LLPS to form gel-like condensates *in vitro* (31, 39). Further, as noted above, the NHA9 (40) and other NUP98 FOs (Supplementary Table S1) were previously shown to localize within nuclear puncta, which we and others (27, 31) propose are aberrant transcriptional condensates. We propose that these NUP98 FO–driven nuclear puncta form through LLPS via a combination of homotypic and heterotypic mechanisms (Fig. 1A). Herein, we present *in vitro* and cellular data showing that both homotypic and heterotypic mechanisms of LLPS are directly linked to the formation of nuclear puncta by NHA9 in cells, which in turn are associated with aberrant transcription and transformation of lineage-negative hematopoietic stem and progenitor cells (lin^−^ HSPC). Further, we show that three additional leukemia-associated NUP98 FOs form nuclear puncta and transform lin^−^ HSPCs, and that nuclear puncta are present in human NUP98-rearranged AML cells.

## Results

### NHA9 Forms Puncta in Cells and *in Vitro* via LLPS

To understand the mechanism driving nuclear puncta formation by NHA9, we expressed monomeric-enhanced GFP (mEGFP)–tagged NHA9 (G-NHA9) in HEK293T cells and quantified the features of the resulting nuclear puncta in many cells. G-NHA9 formed hundreds of puncta in cell nuclei (Fig. 1B) with puncta number increasing with the G-NHA9 nuclear concentration ([G-NHA9]; Fig. 1C; Supplementary Fig. S1A and S1B). Importantly, the mEGFP tag negligibly affected puncta formation (Supplementary Fig. S1C). Fluorescence recovery after photobleaching (FRAP) experiments showed that G-NHA9 molecules are mobile within puncta (Fig. 1D), and puncta showed extensive movement within cell nuclei, but fusion events were not observed (Supplementary Video S1). Average volumes of G-NHA9 puncta (*V_p_*) increased slightly with increasing [G-NHA9] (Fig. 1E). We propose that G-NHA9 puncta form through LLPS driven by interactions mediated by the multivalent FG motifs and interactions of the HOXA9 homeodomain with DNA (Fig. 1A).

We also probed LLPS by NHA9 using *in vitro* assays and observed formation of submicron-size condensates at concentrations ≥10 nmol/L (Fig. 1F). At 20 μmol/L, NHA9 condensates exhibited features characteristic of formation through LLPS (e.g., many exhibited circular morphology apparently driven by surface tension; ref. 41). Turbidity assays also indicated condensate formation (Supplementary Fig. S1D). Notably, FRAP results showed that the NHA9 condensates prepared *in vitro* rapidly transitioned to a gel-like phase, as shown by reduced values of the mobile fraction (*M_f_*) and *T*_1/2_ for NHA9 shortly after induction of condensation (Fig. 1G) and the inability to fuse in time-lapse fluorescence microscopy images (Supplementary Video S1). Together, our findings show that NHA9 forms condensates *in vitro* through the process of homotypic LLPS and suggest that homotypic FG motif interactions contribute to puncta formation in cells.

### DNA Binding by NHA9 Modulates LLPS Behavior

**Figure 2.**
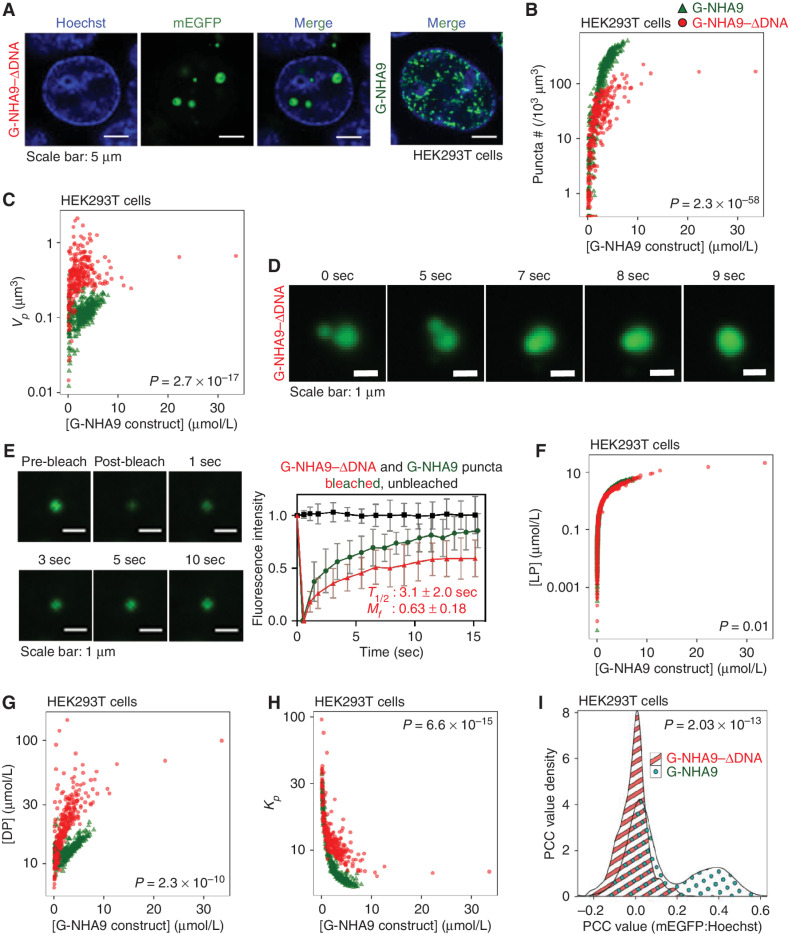
DNA binding by the HOXA9 homeodomain of NHA9 influences puncta morphology and behavior. **A,** Representative confocal microscopy image of live HEK293T cells expressing G-NHA9–ΔDNA (green). DNA is stained with Hoechst dye (blue). An overlay of the G-NHA9–expressing cell from Fig. 1B is included for comparison (right). **B** and **C,** Plots of puncta # (/10^3^ μm^3^; **B**) and *V_p_* (μm^3^; **C**) versus [G-NHA9 construct] for G-NHA9 (green) and G-NHA9–ΔDNA (red) from data represented in **A**. Data are plotted on a semi-log (*y*-axis: log_10_) scale. **D,** Still images of multiple time points taken from a time-lapse confocal fluorescence microscopy video (Supplementary Video S2) of a fusion event in an HEK293T cell expressing G-NHA9–ΔDNA. **E,** Confocal micrographs of FRAP of a G-NHA9–ΔDNA punctum in HEK293T cells at different time points after photobleaching (left). Fluorescence recovery curves are shown for bleached (red, right) and unbleached puncta (black, right). The recovery curve for G-NHA9 is also provided for comparison (green). Individual puncta were manually tracked at different times, and the G-NHA9–ΔDNA fluorescence intensity versus recovery time was plotted as the mean ± SD (*n* = 20). The pairwise *P* value for the recovery curves between G-NHA9 and G-NHA9–ΔDNA is 2.2 × 10^−16^ using the *t* test. **F–H,** Plots of the concentration of the NHA9 construct in the nuclear light phase ([LP], μmol/L; **F**) and within puncta (termed the dense phase; [DP], μmol/L; **G**), and the *K_p_* (${K_p}{\mathrm{\ }} = {\mathrm{\ }}\frac{{[ {{\mathrm{DP}}} ]}}{{[ {{\mathrm{LP}}} ]}}$ (**H**) versus [G-NHA9 construct] for G-NHA9 (green) and G-NHA9–ΔDNA (red). Data are plotted on a semi-log (*y*-axis: log_10_) scale. **I,** 1D-density distribution of PCC per cell for G-NHA9 and G-NHA9–ΔDNA to analyze the linear relationship of the signal between mEGFP and Hoechst. Refer to Supplementary Table S2 for mean values ± standard error. The pairwise *P* values between G-NHA9 and G-NHA9–ΔDNA are shown in each plot (**B**, **C**, **F–I**; see Methods; *n* = 935 and 780 in **B**, **F**, and **I** including the cells with zero punctum, and *n* = 378 and 254 in **C**, **G**, and **H** excluding the cells with zero punctum, respectively, for G-NHA9 and G-NHA9–ΔDNA).

We next asked whether DNA binding influences condensation behavior of G-NHA9 in HEK293T cells by expressing a construct with the homeodomain mutated to weaken DNA binding (G-NHA9–ΔDNA; Supplementary Fig. S2A). Three amino acids were mutated based on analysis of the HOXA9 homeodomain/DNA structure (42) using FoldX (43). In contrast to G-NHA9, G-NHA9–ΔDNA formed a smaller number of large, round condensates localized in both the nucleus and cytoplasm (Fig. 2A and B). The average volumes of nuclear G-NHA9–ΔDNA puncta were 3-fold larger than those for G-NHA9 (Fig. 2C; Supplementary Table S2), and they exhibited fusion events (Fig. 2D; Supplementary Fig. S2B; Supplementary Videos S2 and S3). Similar to G-NHA9, G-NHA9–ΔDNA molecules within condensates were mobile, although the *M_f_* was reduced relative to that for G-NHA9 (Fig. 2E). Additional features differed between the G-NHA9–ΔDNA and G-NHA9 condensates. For example, although values of the average concentration in the nucleoplasm (light phase, LP; [LP]) for G-NHA9 and G-NHA9–ΔDNA were similar (Fig. 2F; Supplementary Table S2), the corresponding values of the average concentration within puncta (dense phase, DP; [DP]) differed (Fig. 2G), with G-NHA9–ΔDNA partitioning (*K_p_*) over 2-fold more than G-NHA9 into puncta (Fig. 2H; Supplementary Table S2). Further, analysis of the Pearson correlation coefficient (PCC) for images of the two constructs showed that the condensates formed by G-NHA9 overlap with DNA to a significantly larger extent than those of G-NHA9–ΔDNA (Fig. 2I). Collectively, these results demonstrate that the puncta-forming behavior of G-NHA9 in HEK293T cells is strongly influenced by binding to DNA.

To evaluate the influence of the C-terminal HOXA9 region on LLPS by NHA9 and NHA9–ΔDNA, we deleted this region, resulting in NUP98-N. NUP98-N formed condensates at concentrations as low as 10 nmol/L *in vitro* (Supplementary Fig. S2C). In HEK293T cells, mEGFP-tagged NUP98-N (G-NUP98-N) formed a smaller number of slightly larger puncta than G-NHA9–ΔDNA and displayed slightly greater partitioning into puncta (1.13-fold; Supplementary Fig. S2D–S2G; Supplementary Table S2). These results indicate that the FG-rich region is the main driver of LLPS by G-NHA9–ΔDNA and that the mutated HOXA9 region in this construct reduces the propensity for LLPS, probably by increasing protein solubility (e.g., due to many charged and polar residues; Supplementary Fig. S2H). This idea is supported by the failure of the HOXA9 region of NHA9 to form condensates *in vitro* (Supplementary Fig. S2I).

Time-lapse imaging of G-NUP98-N puncta in cells revealed that they readily fuse (Supplementary Fig. S2J; Supplementary Video S4), and FRAP analysis showed that G-NUP98-N molecules within them were mobile, as observed for G-NHA9–ΔDNA (Supplementary Fig. S2K). Together, our observations show that the two NHA9–derived constructs that lack DNA-binding activity, G-NHA9–ΔDNA and G-NUP98-N, form condensates in HEK293T cells through LLPS. The observation that G-NHA9 partitions significantly less extensively into small puncta than G-NHA9–ΔDNA and G-NUP98-N do into large puncta (Fig. 2H; Supplementary Fig. S2G) shows that DNA binding by G-NHA9 (a type of heterotypic interaction) competes with its FG motif–dependent interactions, modulating partitioning within puncta. Based upon the circular appearance and diffusivity of mEGFP-tagged molecules within G-NHA9 puncta, and the concordance of the concentration dependence of *K_p_* values for this construct and its DNA binding–deficient counterparts, indicative of multicomponent interactions (44), we conclude that they are liquid-like, chromatin-associated condensates that form through the process of LLPS.

### FG Motif–Dependent Interactions Drive NHA9 LLPS in Cells and *in Vitro*

**Figure 3.**
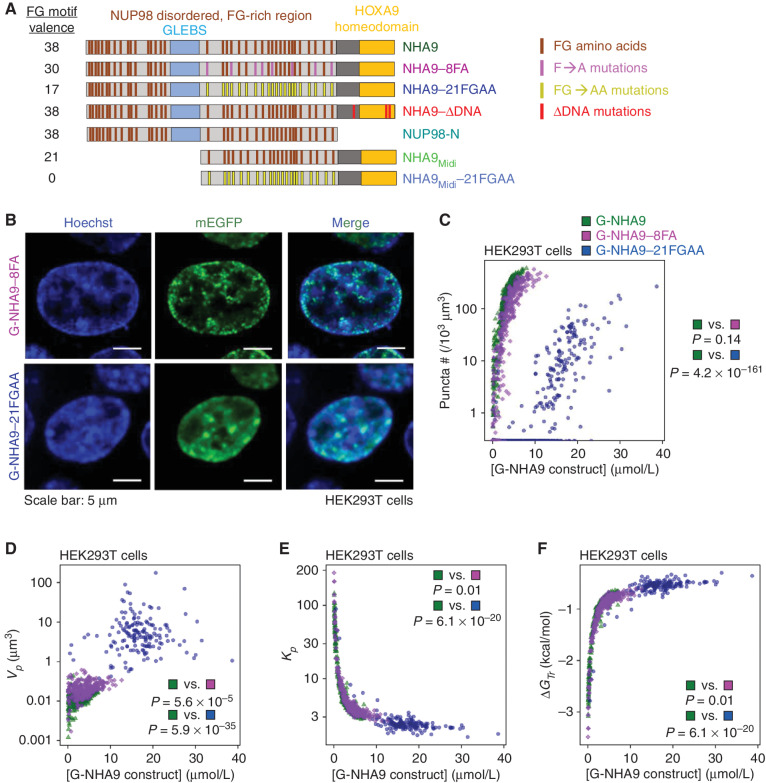
Mutation of multiple F and FG residues in the FG-rich IDR of NHA9 alters puncta formation in cells. **A,** Schematic of NHA9 and mutant constructs used in this study. FG motif valence is shown on the left. **B,** Representative image of live HEK293T cells expressing G-NHA9–8FA (top, green) and G-NHA9–21FGAA (bottom, green). DNA is stained with Hoechst dye (blue). **C–F,** Plots of puncta # (/10^3^ μm^3^; **C**), *V_p_* (μm^3^; **D**), *K_p_* (${K_p}{\mathrm{\ }} = {\mathrm{\ }}\frac{{[ {{\mathrm{DP}}} ]}}{{[ {{\mathrm{LP}}} ]}}$ (**E**), and Δ*G_Tr_* (kcal/mol; **F**) versus [G-NHA9 construct] for G-NHA9 (green), G-NHA9–8FA (purple), and G-NHA9–21FGAA (blue) from data represented in **B**. Data are plotted on a semi-log (*y*-axis: log_10_) scale in **C–E**. Refer to Supplementary Table S2 for mean values ± standard error. The pairwise *P* value between G-NHA9 versus G-NHA9–8FA and G-NHA9 versus G-NHA9–21FGAA is shown in each plot (**C–F**; *n* = 935, 683, and 865 in **C** including the cells with zero punctum and *n* = 378, 273, and 159 in **D–F** excluding the cells with zero punctum, respectively, for G-NHA9, G-NHA9–8FA, and G-NHA9–21FGAA).

Homotypic LLPS of NUP98-N *in vitro* is driven by intermolecular interactions between multivalent FG motifs (ref. 39; Fig. 3A). Our data with G-NUP98-N (Supplementary Fig. S2E–S2G) support that FG motif–dependent interactions contribute to LLPS by G-NHA9 in cells. To further test this, we modified the number of FG motifs within G-NHA9 using mutagenesis and monitored LLPS behavior in HEK293T cells. We generated two mutant constructs with reduced FG motif valence (corresponding to the number of FG motifs; Fig. 3A), one with eight phenylalanine residues mutated to alanine (8FA) and another with 21 phenylalanine–glycine residue pairs mutated to alanine (21FGAA), within the C-terminal region of NUP98-N within NHA9 (Fig. 3A). G-NHA9–8FA formed puncta in cells that were very similar in number and size to those formed by unmutated G-NHA9, whereas G-NHA9–21FGAA formed puncta that were less numerous and larger in size (Fig. 3B–D; Supplementary Table S2). Further, the mutant G-NHA9 constructs partitioned less extensively into puncta (Fig. 3E; Supplementary Table S2). We expressed the differences in *K_p_* values for these constructs in thermodynamic terms by converting them into Gibbs free energy of transfer (Δ*G_Tr_*) values (ref. 44; Fig. 3F; Supplementary Table S2), a measure of the energetic favorability of partitioning into a condensate (ref. 44; negative values are favorable and positive values are unfavorable). The Δ*G_Tr_* value became less negative (less favorable) as the number of FG motifs was reduced (Fig. 3F; Supplementary Table S2), indicating that the valence of these motifs (38 vs. 30 or 17; Fig. 3A) governs partitioning of the G-NHA9 constructs into puncta. We also noted that apparent saturation concentration (*C*_sat_) values (the [G-NHA9 construct] value at which puncta begin to form in cells) increased with decreased FG motif valence (Fig. 3C–F; most apparent in Fig. 3C for G-NHA9 vs. G-NHA9–21FGAA).

We next applied the 21FGAA mutations to a truncated construct spanning the C-terminal portion of NUP98-N within G-NHA9 (termed G-NHA9_Midi_; Fig. 3A), previously reported to transform mouse hematopoietic progenitor cells (23, 45). However, in contrast to constructs studied by others (23, 45), we omitted the GLEBS domain, which mediates protein–protein interactions (17), from NHA9_Midi_ to probe the specific role of its 21 FG motifs in puncta formation. The G-NHA9_Midi_ construct formed a relatively small number of large condensates, while the corresponding 21FGAA mutant displayed primarily diffuse localization (Supplementary Fig. S3A). Cell image analysis showed that G-NHA9_Midi_ formed puncta at lower expression levels that were much smaller (Supplementary Fig. S3B–S3E) than those formed by G-NHA9–21FGAA (Fig. 3C–F; Supplementary Table S2), indicating the importance of FG motif valence in governing LLPS behavior (21 for G-NHA9_Midi_ and 17 for G-NHA9–21FGAA; Fig. 3A). These results demonstrate that the 21 FG motifs within the C-terminal region of NUP98-N, in the context of G-NHA9_Midi_, are necessary and sufficient for nuclear puncta formation and that the GLEBS domain is not required.

We noted that a fraction of puncta for the various G-NHA9 constructs formed at the nuclear periphery (see Figs. 1B and 3B; Supplementary Fig. S3A). The parent protein, NUP98, localizes within the NPC, but the NPC-anchoring domain is absent in NHA9 (12). Immunofluorescence (IF) analysis of an NPC protein (NUP107) in fixed HEK293T cells expressing G-NHA9 showed localization immediately outside the nuclear periphery (defined by the DAPI DNA stain; Supplementary Fig. S3F), a region lacking G-NHA9 puncta. Further, only a small fraction of G-NHA9 puncta were localized near the nuclear periphery (Supplementary Fig. S3G). Therefore, we conclude that the small fraction of puncta we observed at the nuclear periphery are not colocalized with NPCs.

**Figure 4.**
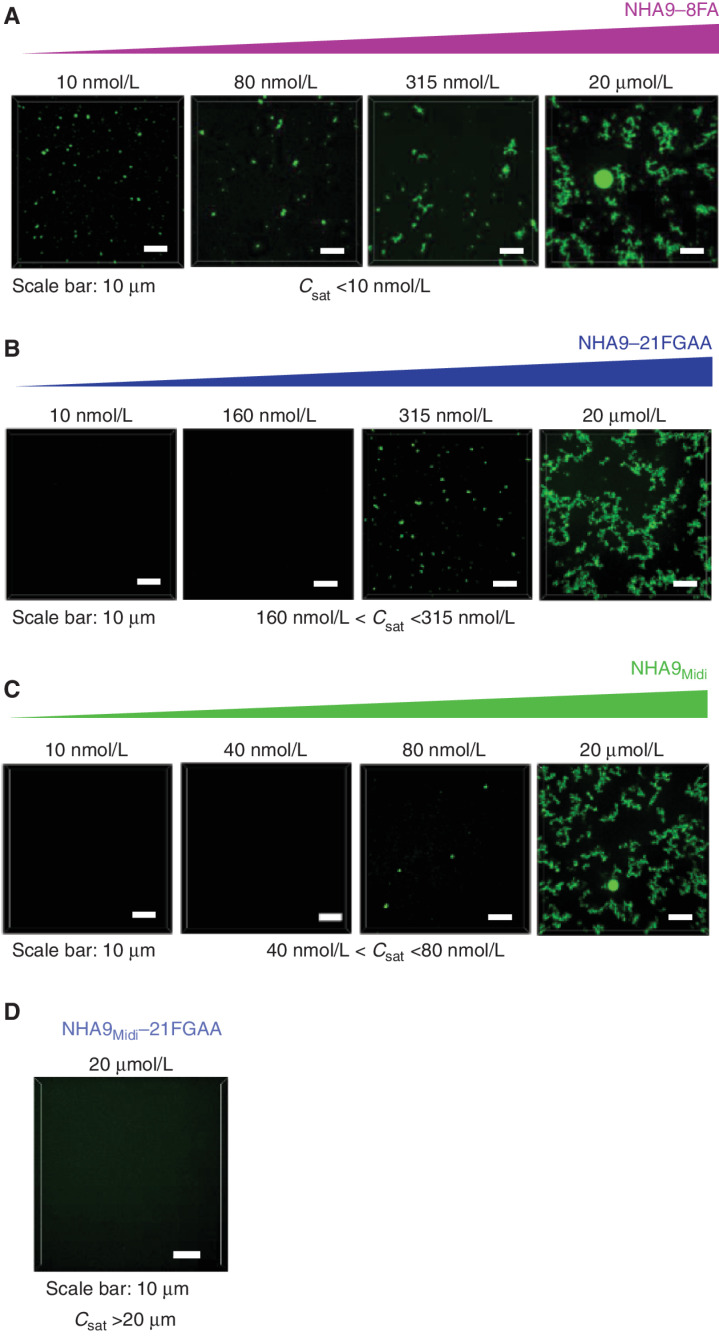
Mutation of F and FG residues in the FG-rich IDR of NHA9 alters LLPS behavior *in vitro*. **A–C,** Confocal fluorescence micrographs of Alexa 488–labeled NHA9–8FA (**A**), NHA9–21FGAA (**B**), and NHA9_Midi_ (**C**) condensates *in vitro* with increasing protein concentration. Micrographs are presented as maximum intensity projections of 13 Z-stack images acquired over 6 μm with 0.5 μm resolution. **D,** Confocal micrograph of Alexa 488–labeled NHA9_Midi_–21FGAA at a concentration of 20 μmol/L. Saturation concentration (*C*_sat_) is less than 10 nmol/L (**A**), between 160 nmol/L and 315 nmol/L (**B**), and between 40 nmol/L and 80 nmol/L (**C**).

We next asked whether mutations to FG motifs within G-NHA9 and G-NHA9_Midi_ would affect homotypic LLPS *in vitro*. NHA9–8FA formed condensates at a *C*_sat_ value similar to that for NHA9 (*C*_sat_ <10 nmol/L), whereas NHA9–21FGAA did so only above 160 nmol/L (Fig. 4A and B; Supplementary Fig. S4A–S4C). NHA9_Midi_ formed condensates between 40 and 80 nmol/L, whereas NHA9_Midi_–21FGAA did not form condensates up to 20 μmol/L (Fig. 4C and D; Supplementary Fig. S4D). Turbidity assays yielded similar differences in the condensation behavior of these constructs (Supplementary Fig. S4E). Importantly, our *in vitro* results with these constructs closely parallel those obtained with the corresponding mEGFP-tagged constructs in HEK293T cells. These observations demonstrate that interactions mediated by multivalent FG motifs contribute to the LLPS behavior of NHA9 and NHA9_Midi_ in HEK293T cells. In our *in vitro* assays, only homotypic interactions involving the FG motifs are possible. However, in cells, both homotypic and heterotypic protein–protein interactions involving these residues are possible. In addition to FG motif–mediated interactions, however, additional heterotypic interactions with DNA mediated by the HOXA9 homeodomain influence LLPS (Fig. 1A), driving the formation of many small, chromatin-associated nuclear puncta by G-NHA9 in HEK293T cells.

### Condensation Behavior of NHA9 Variants in lin^−^ HSPCs

**Figure 5.**
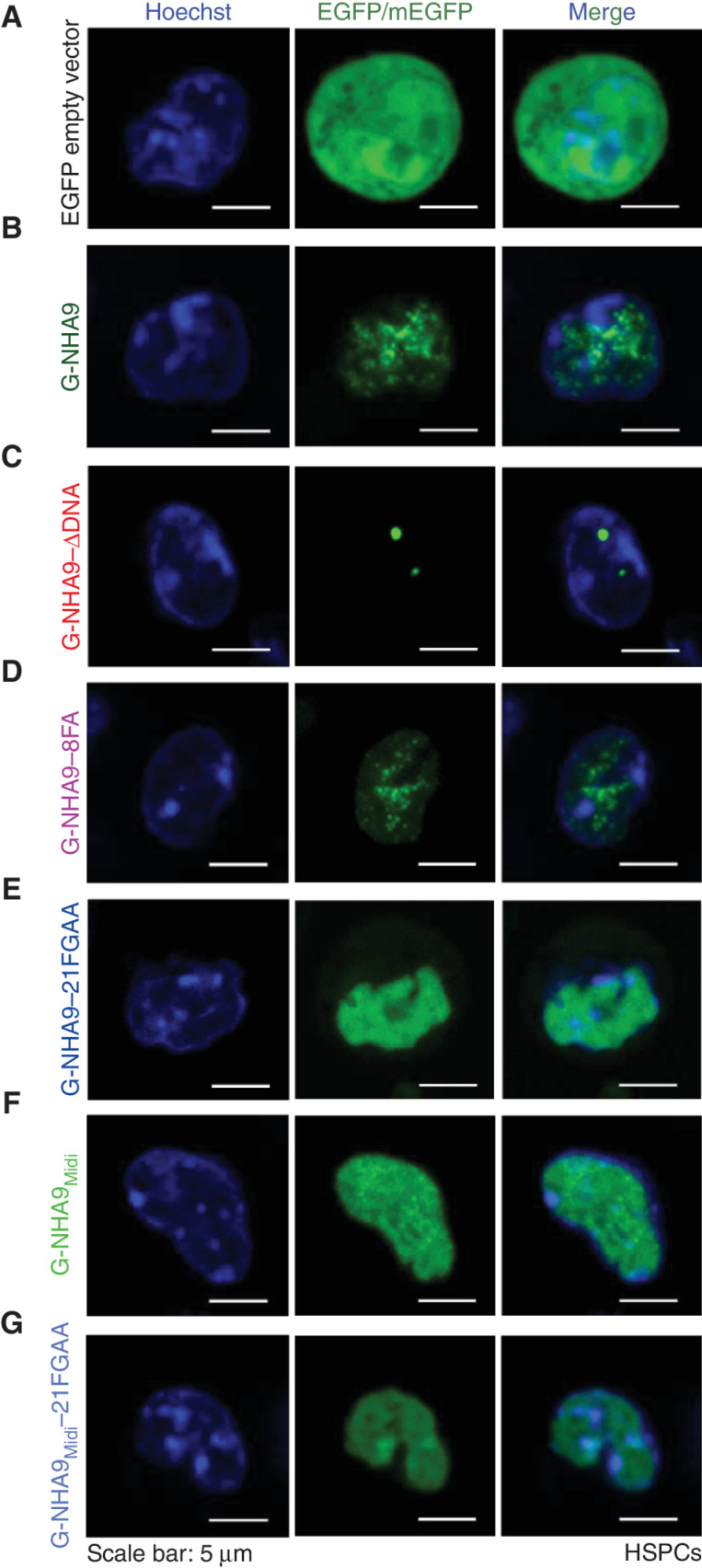
The NHA9 constructs form puncta in lin^−^ HSPCs. **A–G,** Representative images of live lin^−^ HSPCs expressing EGFP empty vector as a control (**A**), G-NHA9 (**B**), G-NHA9–ΔDNA (**C**), G-NHA9–8FA (**D**), G-NHA9–21FGAA (**E**), G-NHA9_Midi_ (**F**), and G-NHA9_Midi_–21FGAA (**G**).

To address the relevance of our LLPS findings in HEK293T cells to leukemia, we performed studies using primary mouse lin^−^ HSPCs, a model system known to faithfully recapitulate the leukemogenic effects of NUP98 FOs seen in human hematopoietic cells. For example, previous studies have shown that transplantation of lin^−^ HSPCs expressing NHA9 and other NUP98 FOs into recipient mice results in the development of myeloid leukemia (8, 21, 22, 46). Lin^−^ HSPCs infected with lentiviral expression vectors were isolated by FACS 2 days after viral infection and imaged after 24 to 48 hours. The G-NHA9 construct displayed dynamic nuclear puncta (Fig. 5A and B; Supplementary Video S5) qualitatively similar in number and size to those observed in HEK293T cells (Fig. 1B). The puncta observed for G-NHA9–ΔDNA were sparser, larger, and denser than those observed for G-NHA9, as observed in HEK293T cells (Fig. 5B and C; Supplementary Fig. S5A). Further, the puncta-forming behavior of the other full-length NHA9 constructs examined paralleled that observed in HEK293T cells (Fig. 5D–G; Supplementary Fig. S5B and S5C), although the expression levels for the full range of G-NHA9 constructs were lower in HSPCs than in HEK293T cells [compare the range of G-NHA9 construct concentration values observed in HEK293T cells (Figs. 1C and E; 2B, C, and F–H; 3C–F) with those seen in lin^−^ HSPCs (Supplementary Fig. S5A–S5C)]. As in HEK293T cells, only a small percentage of puncta formed by G-NHA9 and variant constructs were localized at the nuclear periphery in lin^−^ HSPCs (Supplementary Fig. S5D). Importantly, these results demonstrate that puncta-forming behavior is an intrinsic property of the NHA9 constructs studied, and that formation of many small nuclear puncta by G-NHA9 is driven by interactions mediated by both multivalent FG motifs and the HOXA9 homeodomain.

### NHA9 Transforms Lin^−^ HSPCs and Alters Gene Expression

**Figure 6.**
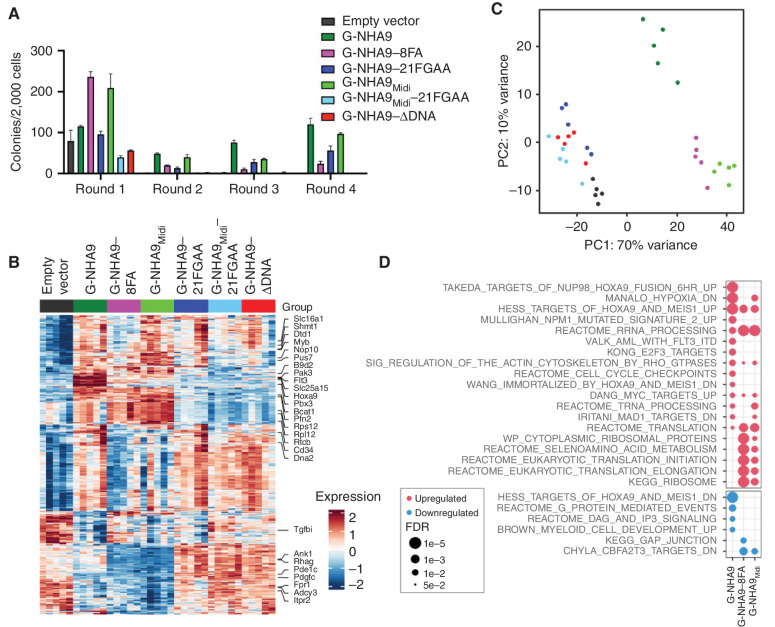
Expression of high FG motif valence NHA9 constructs in lin^−^ HSPCs leads to hematopoietic cell transformation and aberrant expression of *Hox* family and other genes. **A,** Average number of colonies per 2,000 cells for lin^−^ HSPCs expressing negative control empty vector and mEGFP-tagged NHA9 and mutant constructs. The values of colony numbers shown are mean ± SD from triplicate technical replicates of a representative experiment. **B–D,** RNA-seq was performed for lin^−^ HSPCs expressing empty vector, G-NHA9, or mutants after 1 week of growth in methylcellulose containing myeloid and erythroid growth factors (*n* = 5 for each condition). **B,** Heat map for differentially expressed genes of interest. **C,** PCA of the 500 most variable genes. **D,** Gene set enrichment analysis for cells expressing G-NHA9, G-NHA9–8FA, or G-NHA9_Midi_—each versus empty vector. Pathways of interest are shown, with a complete list of significantly upregulated or downregulated gene sets in Supplementary Table S3. The most significantly dysregulated genes from each pathway are marked in **B**.

We next examined whether the FG motif valence or DNA-binding activity of the NHA9 constructs and their puncta-forming behavior were correlated with transformation as measured in colony-forming unit assays of NUP98 FO-transduced lin^−^ HSPCs (Supplementary Fig. S6A). Expression of either the G-NHA9, G-NHA9–8FA, G-NHA9–21FGAA, or G-NHA9_Midi_ constructs induced serial replating of lin^−^ HSPCs (Fig. 6A; Supplementary Fig. S6B), whereas G-NHA9_Midi_–21FGAA and G-NHA9–ΔDNA did not sustain self-renewal (Fig. 6A). However, although expression of G-NHA9, G-NHA9–8FA, and G-NHA9_Midi_ induced myeloid lineage differentiation, including expression of CD11b and Gr1, G-NHA9–21FGAA did not (Supplementary Fig. S6C). These results suggest that only NHA9 constructs with the highest FG motif valence values (Fig. 3A) drive transformation, as evidenced by a myeloid serial replating phenotype. Our results with G-NHA9_Midi_ show that the 21 FG motifs within the C-terminal region of NUP98-N are important contributors to induction of the myeloid phenotype in transformed HSPCs, although the loss of eight of these motifs was compensated by the 17 N-terminal FG motifs within the G-NHA9–8FA construct. Further, both high-valence FG motifs and direct DNA binding by the HOXA9 homeodomain are required for HSPC transformation, as shown by the loss of this ability with G-NHA9–ΔDNA. Collectively, our results strongly suggest that LLPS by NHA9 contributes to the transformation of lin^−^ HSPCs and to leukemogenesis in AML.

NUP98 FOs, including NHA9, bind to *HOX* family and other genes in hematopoietic stem cells (23), driving aberrant expression of these genes (6, 7, 25) and cell transformation (23). To determine whether the LLPS propensity of the G-NHA9 constructs, as quantified by FG motif valence, was correlated with patterns of gene expression, we performed RNA sequencing (RNA-seq) of lin^−^ HSPCs transduced with empty vector, G-NHA9, G-NHA9–8FA, G-NHA9–21FGAA, G-NHA9_Midi_, G-NHA9_Midi_–21FGAA, and G-NHA9–ΔDNA. Cells for RNA-seq analysis were harvested from the first round of plating. The results showed that G-NHA9 induced expression of *Hox* and other genes (Fig. 6B; Supplementary Fig. S6D) whose overexpression was previously shown to be associated with cell transformation and leukemogenesis in mice and humans (23, 25, 26). G-NHA9–8FA and G-NHA9_Midi_, but not the other constructs tested, also induced expression of *Hox* genes (Fig. 6B; Supplementary Fig. S6D). Principal component analysis (PCA; Fig. 6C) showed that the three myeloid phenotype–inducing NHA9 constructs induced gene expression profiles in lin^−^ HSPCs that were different from those induced by G-NHA9–21FGAA, G-NHA9_Midi_–21FGAA, G-NHA9–ΔDNA, and the empty vector control (Fig. 6B).

The results above show that transformation of lin^−^ HSPCs by G-NHA9, G-NHA9–8FA, and G-NHA9_Midi_ is associated with upregulation of a common set of developmental regulatory genes. Several genes, including *Hoxa9* and *Flt3*, were upregulated by the three myeloid lineage–inducing NUP98 FOs but not by the others with a homeodomain, suggesting that they are significant contributors to induction of the myeloid phenotype in lin^−^ HSPCs (Fig. 6B; Supplementary Fig. S6D). Other developmental genes, including *Meis1* and *Pbx3*, however, were strongly upregulated in G-NHA9 cells but not in the G-NHA9–8FA, G-NHA9_Midi_, or other nontransforming conditions (Supplementary Fig. S6D). These genes then may be dispensable for myeloid cell transformation in NHA9 construct–expressing cells. Gene set enrichment analysis (Fig. 6D; Supplementary Table S3) showed that although G-NHA9 shared many target genes with G-NHA9–8FA and G-NHA9_Midi_, the latter FOs led to increased expression of unique gene sets. Many such genes, including *Rps12* and *Rpl12*, encode proteins with functions related to the ribosome and translation (Fig. 6B and D). Nevertheless, all three of these G-NHA9 constructs robustly form nuclear puncta in HSPCs (Fig. 5B, D, and F; Supplementary Fig. S5A–S5C; Supplementary Video S5) and HEK293T cells (Figs. 1 and 3; Supplementary Fig. S3), linking this type of LLPS behavior with increased self-renewal activity and myeloid differentiation in lin^−^ HSPCs.

Additional NUP98 FOs Undergo LLPS and Transform Hematopoietic Cells

**Figure 7.**
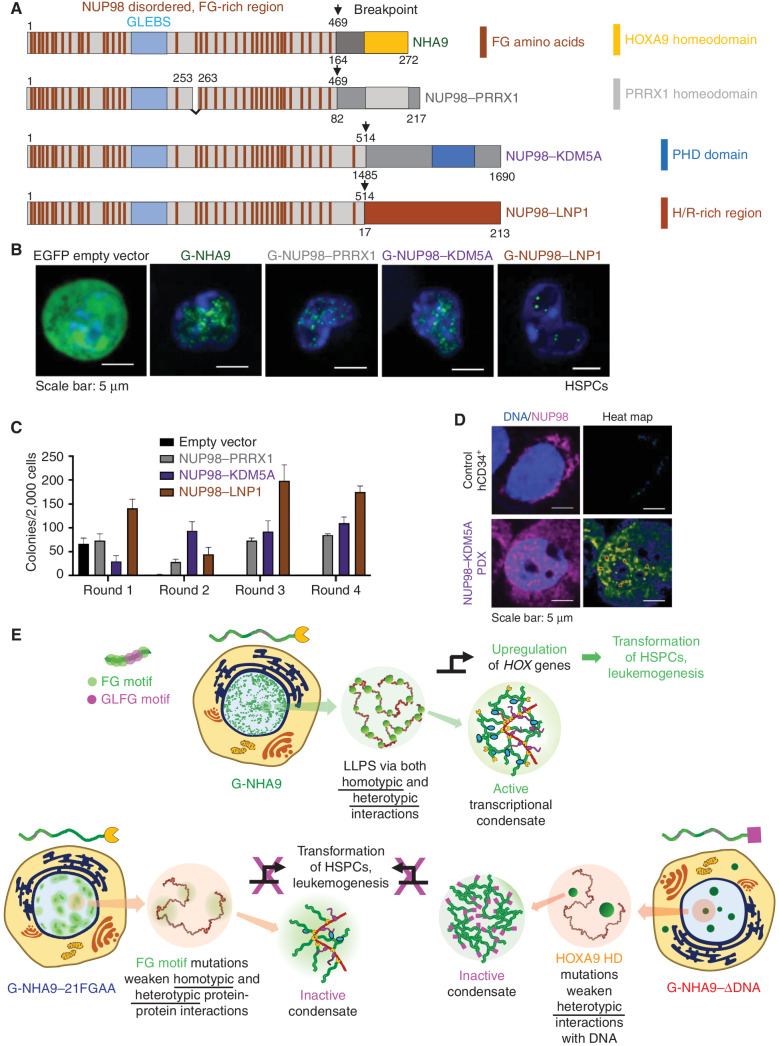
Additional leukemia-associated NUP98 FOs form nuclear puncta and transform lin^−^ HSPCs. **A,** Schematic of NHA9 and three additional NUP98 FOs (NUP98–PRRX1, NUP98–KDM5A, and NUP98–LNP1). Numbers indicate the amino acid residue. Numbers above the schematic reflect NUP98 residues, whereas numbers beneath reflect the fusion partner's residues. **B,** Representative images of live lin^−^ HSPCs expressing G-NUP98–PRRX1, G-NUP98–KDM5A, and G-NUP98–LNP1. EGFP empty vector and G-NHA9 are included for comparison. **C,** Average number of colonies per 2,000 cells for lin^−^ HSPCs expressing negative control empty vector and NUP98 FOs. Data shown are mean ± SD from triplicate technical replicates of a representative experiment. **D,** Representative images of a fixed, nontransduced human CD34^+^ (hCD34^+^) cell (top) and a NUP98–KDM5A PDX cell (bottom) stained with an antibody against NUP98. NUP98 is magenta, and DNA is blue. The heat map is a normalized representation of NUP98 fluorescence intensity across the PDX cell. **E,** Conceptual scheme illustrating how LLPS by NHA9 mediates the formation of aberrant transcriptional condensates in hematopoietic cells. NHA9 (top, left cell image) undergoes LLPS to form many small, chromatin-associated puncta that drive aberrant expression of *Hox* and other genes and transform hematopoietic cells. LLPS is driven by both homotypic and heterotypic interactions. Mutation of FG motifs (NHA9–21FGAA, bottom left) weakens both homotypic and heterotypic protein–protein interactions, yielding less numerous, larger, and less dense puncta that do not activate *Hox* gene expression or transform HSPCs. Mutation of residues in the HOXA9 homeodomain (HD; NHA9–ΔDNA, bottom right) weakens heterotypic interactions with DNA, yielding less numerous, larger, and more dense puncta that also do not induce the leukemogenic phenotype in HSPCs.

We next tested whether additional NUP98 FOs associated with acute leukemia formed liquid-like condensates in HEK293T cells and lin^−^ HSPCs (Fig. 7A), including one displaying an alternative DNA-binding homeodomain (NUP98–PRRX1; ref. 47), another with an epigenetic mark–binding PHD domain (NUP98–KDM5A; ref. 21), and a third displaying a histidine and arginine (H/R)–rich region of unknown structure and function (NUP98–LNP1; ref. 48). We expressed mEGFP-tagged forms of these NUP98 FOs in HEK293T cells and found that all three formed nuclear puncta (Supplementary Fig. S7A–S7E), although they displayed a range of features (Supplementary Fig. S7F). The puncta formed by G-NUP98–PRRX1, including their number, volume, and *K_p_* values, were very similar to those for G-NHA9 (Supplementary Fig. S7B, S7C, and S7F; Supplementary Table S2), likely because the two homeodomains bind DNA with similar affinity. G-NUP98–KDM5A, which binds histone H3 displaying dimethyl and trimethyl lysine 4 (H3K4-me2/3) epigenetic marks within chromatin (21), formed larger, less numerous puncta at generally higher expression levels than G-NHA9 (Supplementary Fig. S7D and S7F; Supplementary Table S2). Finally, G-NUP98–LNP1 formed slightly larger puncta than G-NHA9 (Supplementary Fig. S7E and S7F; Supplementary Table S2) and showed greater partitioning into puncta than the other three NUP98 FOs (Supplementary Fig. S7F; Supplementary Table S2). As for G-NHA9, the mEGFP-tagged molecules within the puncta discussed above were dynamic (Supplementary Fig. S7G–S7I). Additionally, for G-NUP98–LNP1, we observed puncta fusion (Supplementary Fig. S7J; Supplementary Video S6). Together, these results support that the puncta formed by these three additional NUP98 FOs in HEK293T cells reflect the liquid-like features characterized in detail for G-NHA9 and suggest that this is a universal feature of NUP98 FOs.

To determine if the additional NUP98 FOs behave similarly in hematopoietic cells, we expressed them in lin^−^ HSPCs using lentivirus and performed imaging after 24 hours. All three FOs formed puncta that were very similar to those seen in HEK293T cells (Fig. 7B), although the cellular expression levels were at least 10-fold lower in HSPCs than in HEK293T cells (Supplementary Fig. S7K). The low expression levels precluded accurate quantitation of puncta features. However, these results show that, despite lower expression levels, all four NUP98 FOs tested formed apparently liquid-like puncta in both HEK293T cells and lin^−^ HSPCs.

Next, we determined whether the additional NUP98 FOs transform cells as measured by the colony-forming unit assay in lin^−^ HSPCs. Expression of each FO resulted in sustained colony formation through four rounds of replating (Fig. 7C). Analysis of differentiation markers showed that the HSPCs transformed by the three additional NUP98 FOs exhibited the myeloid phenotype (Supplementary Fig. S7L). These findings support that the relationship between puncta formation driven by LLPS and hematopoietic cell transformation seen with G-NHA9 is common to these three additional FOs, and possibly all NUP98 FOs due to their conservation of the LLPS-prone N-terminal FG motif–rich region of NUP98 and C-terminal regions that often display identifiable DNA- and/or chromatin-binding domains (ref. 18; Supplementary Table S1).

To address clinical relevance, we obtained hematopoietic cells from an AML patient-derived xenograft (PDX; Supplementary Fig. S7M) harboring the *NUP98–KDM5A* fusion oncogene (49). IF analysis of the PDX cells using a monoclonal antibody against NUP98 revealed nuclear puncta (Fig. 7D) qualitatively similar in size and number to those observed in mouse lin^−^ HSPCs (Fig. 7B). Nuclear puncta were not observed in identically stained nontransduced human CD34-positive HSPCs (hCD34^+^ cells; Fig. 7D). Due to the scarcity of cells and high background staining in the IF images, we were unable to quantify puncta features for the human PDX cells harboring the *NUP98–KDM5A* fusion oncogene. However, we were able to detect puncta in 10 cells (Supplementary Fig. S7N). To minimize artifacts due to antibody reactivity with endogenous NUP98 within NPCs, we excluded IF intensity within 0.5 μm of the nuclear periphery. This analysis revealed nuclear puncta in several of the PDX cells (Supplementary Fig. S7N and S7O). A similar analysis of control hCD34^+^ HSPCs registered very few puncta (Supplementary Fig. S7N and S7O). Together, these results demonstrate that the NUP98–KDM5A FO forms nuclear puncta in patient-derived AML cells similar to those observed after viral transduction of mouse lin^−^ HSPCs. Broadly, these results support our proposal that formation of nuclear puncta by NUP98 FOs drives aberrant gene expression, hematopoietic cell transformation, and the development of human AML.

## Discussion

Our results show that NHA9 forms hundreds of nuclear puncta through both homotypic and heterotypic mechanisms of LLPS, which we demonstrate is causally linked with aberrant gene expression and transformation of mouse hematopoietic cells to the myeloid lineage. Three additional NUP98 FOs, NUP98–PRRX1, NUP98–KDM5A, and NUP98–LNP1, also form nuclear puncta with apparent liquid-like features and transform and induce myeloid differentiation in mouse lin^−^ HSPCs. Further, the NUP98–KDM5A FO expressed in AML PDX hematopoietic cells formed puncta similar to those observed in mouse lin^−^ HSPCs. Together, our results for multiple NUP98 FOs and those of others for NHA9 (31) demonstrate that LLPS drives the formation of nuclear puncta by these FOs that mediate aberrant gene expression, transform both mouse and human hematopoietic cells, and promote leukemogenesis. The observation of nuclear puncta for many additional AML-associated NUP98 FOs that display conserved domain features (Supplementary Table S1) strongly suggests that homotypic and heterotypic LLPS drives leukemogenesis by most NUP98 FOs.

The ability to undergo both homotypic and heterotypic interactions is encoded in the domain features of NUP98 FOs. Homotypic and heterotypic protein–protein interactions are mediated by the 38 FG motifs within the N-terminal disordered region of NUP98, whereas, in the case of NHA9, additional heterotypic interactions with DNA are mediated by the C-terminal HOXA9 homeodomain (Fig. 7E, top). Together, these interactions drive formation of DNA-associated NHA9 puncta through LLPS. This is supported by the observation of liquid-like features for G-NHA9 (Fig. 1D), as well as for G-NHA9 constructs defective in DNA binding [Figs. 2D and E and 7E (bottom, right); Supplementary Fig. S2B, S2J, and S2K; Supplementary Videos S2 and S3]. The observation of large, round condensates for NHA9 at high concentrations *in vitro* (Fig. 1F) further supports formation through LLPS. Although *in vitr*o NHA9 condensates are gel-like (Fig. 1G), the puncta observed for G-NHA9 in cells are liquid-like, which we propose is due to dynamic heterotypic interactions with many protein partners (27, 31). The strong concentration dependence of *K_p_* values for G-NHA9 and G-NHA9–ΔDNA in HEK293T cells (Fig. 2H) provides strong evidence for puncta formation through multicomponent, heterotypic LLPS (44). Further, the responses of the puncta-forming behavior of the NHA9 constructs to mutations that reduce their FG motif valence are in accord with expectations with LLPS as the driving mechanism. In summary, multiple lines of evidence demonstrate that LLPS is the driving mechanism of NHA9 puncta formation in cells, as discussed by others (31).

Our results demonstrate that the C-terminal FG motif region within NHA9 is an important determinant of puncta-forming behavior, aberrant gene expression, and hematopoietic cell transformation. Supporting this, puncta-forming behavior was severely attenuated and aberrant gene expression and hematopoietic cell transformation were abrogated when only the N-terminal FG motif repeat region is present within NHA9 (as in G-NHA9–21FGAA; Fig. 7E, bottom left). The C-terminal FG motif repeat region not only is longer than the N-terminal region, but also displays higher FG motif valence (21 vs. 17, with six vs. three GLFG motifs) and displays an approximately 80-residue-long prion-like domain (Supplementary Fig. S8). Finally, in addition to enrichment in FGs, the C-terminal region is moderately enriched in asparagine, glutamine, serine, and threonine residues (Supplementary Fig. S8; compare bottom left with bottom right), which can contribute to LLPS (50). The strong contribution of the C-terminal FG motif region to LLPS-driven transformation of lin^−^ HSPCs may explain why virtually all reported NUP98 FOs retain all of the NUP98-N FG motifs (18). It follows logically that gene translocations with breakpoints closer to the 5′ end of the *NUP98* gene, leading to FOs with incomplete C-terminal FG motif regions, would experience blunted LLPS and fail to induce aberrant gene expression and transform hematopoietic cells. In summary, our data support that the C-terminal FG motif region within the NHA9 FO drives LLPS in cell nuclei and contributes to aberrant *Hox* gene expression and transformation of lin^−^ HSPCs.

Finally, we propose that, based on our mechanistic understanding of the LLPS behavior of the four NUP98 FOs we studied, virtually all additional NUP98 FOs, which retain the LLPS-prone FG-rich IDR of NUP98 and DNA- or chromatin-binding domains from the C-terminal fusion partners (18), similarly undergo LLPS, forming nuclear puncta that drive aberrant gene expression and often transform hematopoietic cells. For example, in addition to DNA-binding homeodomains, HMG and other DNA-binding domains are represented within NUP98 FOs, as well as other chromatin-binding domains, including PHD domains, bromodomains, and SET and IQ domains (18). Further, other types of FOs associated with a wide range of cancers contain fused regions of transcription factors (51) that are understood to contain disordered regions prone to LLPS (32). In fact, several reports have indicated that LLPS might play a role in aberrant gene expression linked to leukemogenesis in the case of EWS–FLI1, a driver in Ewing sarcoma, and other FUS/EWS/TAF15 protein family FOs (35, 52, 53). We propose that many FOs, which display the LLPS-prone IDR/chromatin-binding domain organization discussed above, acquire oncogenic function through deleterious functional synergy between their LLPS-prone IDRs and folded chromatin-binding domains (54). This synergy may be responsible for the protein interaction network rewiring associated with FOs (55). Our findings with the four NUP98 FOs reported herein suggest that their leukemogenic effects in hematopoietic cells are rooted in synergy between homotypic and heterotypic interactions that drive LLPS. Critical for the future will be to leverage this understanding to develop targeted approaches to therapeutically modulate aberrant LLPS by FOs (56) and possibly counteract their oncogenic phenotypes.

## Methods

### Cell Culture and Transient Transfections

HEK293T cells (ATCC; RRID:CVCL_0063) were cultured in DMEM with high glucose (Gibco) and supplemented with 1× penicillin/streptomycin (Gibco), 10% FBS (HyClone), and 6 mmol/L l-glutamine (Gibco) and maintained at 37°C in 5% CO_2_. Cells were tested for *Mycoplasma* every 2 months using PCR (e-Myco plus, LiLiF). Cells were authenticated by short tandem repeat profiling (PowerPlex Fusion at the St. Jude Hartwell Center). Cells were transfected in a 96-well plate with 100 ng of plasmid DNA in the CL20 vector backbone using FuGENE HD (Promega) per the manufacturer's instructions. All fusion proteins were N-terminally tagged with monomeric EGFP (A207K mutation in EGFP), and EGFP was used for the empty vector control plasmids (see plasmid list in Supplementary Table S4 for sequences). Cells were used for a maximum of 25 passages after thawing.

### Immunofluorescence

For NPC staining in HEK293T, cells were fixed with 4% paraformaldehyde (Electron Microscopy Sciences) and then incubated for 5 minutes in 0.5% Triton-X-PBS for cell permeabilization. For fixation of hematopoietic cells and NUP98–KDM5A PDX cells, a cytocentrifuge was used to adhere cells to a glass slide by spinning at 400 rpm for 4 minutes. The cells were then rapidly rinsed in 1× PBS–5 mmol/L EGTA, followed by incubation at −20°C in 95% methanol–5 mmol/L EGTA for 30 minutes. The primary antibodies used were mouse anti-NUP107 (Abcam, Mab414, RRID:AB_448181, 1:300) and rat anti-NUP98 (GeneTex, 2H10, RRID: AB_2894964, 1:200). The secondary antibodies used were raised in donkey and conjugated to Alexa Fluor Rhodamine Red-X or Alexa Fluor 647 (Jackson ImmunoResearch; RRID:AB_2340614) at 1:300 diluted in 5% normal donkey serum. Cells were counterstained with DAPI (Invitrogen) diluted in PBS (300 nmol/L) for 2 minutes and then mounted onto glass slides with antifade solution (90% glycerol, 0.5% N-propyl gallate). See Supplementary Methods for additional details.

### Recombinant Protein Expression, Purification, and Labeling

Recombinant proteins were expressed in *Escherichia coli* BL21(DE3) cells (Novagen) using constructs in the pET28-12xHis backbone (see plasmid list in Supplementary Table S4). Proteins were purified using Ni-NTA affinity chromatography, followed by proteolytic removal of the polyhistidine tag. The 12xHis-TEV was removed using Ni-NTA beads, and the flow-through fractions containing cleaved proteins were loaded on an HPLC column (PLRP-S 1000A 8 μmol/L, Agilent Technologies) for the final purification step. Proteins were fluorescently labeled using either maleimide or succini-midyl ester derivatives of Alexa Fluor dyes (Thermo Fisher Scientific) according to the manufacturer's protocol. The labeled protein concentration and efficiency of labeling were determined according to the manufacturer's protocol. See Supplementary Information for additional details.

### *In Vitro* Confocal Microscopy

For constructing phase diagrams, images were acquired using a 3i Marianas spinning disk confocal microscope with SlideBook 6.0 software (3i) and a 63× oil immersion objective, numerical aperture 1.4. The unlabeled NHA9 dissolved in water with 5 mmol/L DTT was mixed with Alexa Fluor–labeled NHA9 dissolved in the same solvent and diluted with 2× PBS (1× PBS; 136.9 mmol/L NaCl, 2.68 mmol/L KCl, 10 mmol/L Na_2_HPO_4_, 1.7 mmol/L KCl, pH 7.4) at 1:1 to induce LLPS. The samples were incubated at room temperature overnight before imaging in a 384-well SensoPlate (Greiner Bio-One) coated with Sigmacote (Sigma-Aldrich), followed by 1% weight/volume pluronic F-127 (Sigma-Aldrich). As the condensates settled to the bottom surface of the coverslip due to gravity, all images were acquired from the bottom of the coverslip to a 6 μm height with a 0.5-μm interval. Images were processed as maximum intensity projections over the different z-slices.

### Fluorescence Recovery after Photobleaching

FRAP experiments were performed on a Marianas spinning disk confocal microscope with SlideBook 6.0 software (3i), using 100× oil immersion objective, numerical aperture 1.45. A circular region of interest (ROI) of diameter 0.5 μm was located at the center of droplets and photobleached to 50% intensity by illuminating the ROI with an appropriate laser. Laser power was set as needed for 100 ms exposure. Background signal was subtracted from the measured fluorescence recovery values and normalized against an unbleached condensate. As NHA9 condensates underwent rapid gelation under *in vitro* conditions, the FRAP experiments were performed within 2, 4, and 8 minutes of the formation of the condensates. For in-cell FRAP, a single punctum was bleached with 50 ms exposure, and the recovery of the fluorescence was tracked manually by shifting a 0.6-μm diameter ROI. The data were normalized against the overall photobleaching for the mEGFP fluorescence. The FRAP data curves were fitted using the simple exponential equation fit given below.




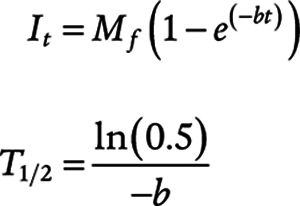




Where *I_t_* is the measured fluorescence intensity at time point *t*, *T*_1/2_ is the half-time of recovery, and *M_f_* is the mobile fraction.

### Turbidity Assays

For all turbidity measurements, different concentrations of the untagged-NHA9 constructs were diluted with 2× PBS, such that the final buffer was 1× PBS. Absorption at 340 nm was measured after 30 minutes of incubation of the samples using a NanoDrop 2000c spectrophotometer (Thermo Fisher Scientific). The 0 μmol/L protein concentration, which is the buffer-only sample, was used as a background, and that buffer-only A_340_ value was subtracted from all the protein A_340_ values. The mean and SD values were obtained from at least three independent sets of experiments.

### Preparation of HSPCs

All mice were maintained in the Animal Resource Center at St Jude Children's Research Hospital and were treated ethically under Animal Care and Use Committee–approved protocol number 584-100669-10/20. Whole bone marrow was harvested from the forelimbs and hindlimbs of male and female 6- to 12-week-old C57Bl/6 mice (RRID: IMSR_JAX:000664) in PBS + 5% FBS (HyClone). Lin^−^ HSPCs were selected using the EasySep Mouse Hematopoietic Progenitor Cell Isolation Kit (STEMCELL Technologies) according to the manufacturer's instructions. Cells were resuspended in IMDM (Gibco) containing 20% FBS (HyClone), 1× penicillin–streptomycin–glutamine (Gibco), 50 ng/mL SCF (PeproTech), 40 ng/mL Flt3 (PeproTech), 30 ng/mL IL6 (PeproTech), 20 ng/mL IL3 (PeproTech), and 10 ng/mL IL7 (PeproTech). Cells were then seeded onto retronectin-treated plates preloaded with virus. Lentiboost B (Sirion Biotech) was added at 1:100 into the cell suspension, and the plates were spun at 800 × *g* and 4°C for 90 minutes. Following this spin, cells were moved to a 37°C incubator and cultured for 2 days. Cells were then collected using cell dissociation buffer (Gibco). FACS was used to isolate mCherry- and/or mEGFP-expressing cells.

### Virus Preparation

Virus was prepared by cotransfection of approximately 40% confluent HEK293T cells grown in DMEM (Lonza) containing 10% FBS (HyClone) and 1× penicillin–streptomycin–glutamine (Gibco). Five-hundred microliters OptiMEM-reduced serum medium (Gibco) was mixed with 40 μL FuGENE HD (Promega) for each 10-cm dish of HEK293T cells. After a 5-minute incubation at room temperature, plasmids were added. The St. Jude vector core prepared all lentiviral virus with constructs in the CCLMPC vector backbone using VSV-G pseudotyping, followed by purification and concentration by ion exchange. For retrovirus, 10 μg plasmid of interest in MSCV backbone + 1 μg of CAG-ECO and 3 μg of gag-pol were added. See plasmid list in Supplementary Table S4. After a 20- to 30-minute incubation at room temperature, OptiMEM mixture was added drop-wise to cells. The media were changed 18 to 24 hours after transfection, and viral supernatants were collected 48 to 72 hours after transfection. Lentivirus was used in the colony-forming unit assay, RNA-seq experiments, and HSPC imaging for all NHA9 constructs. Lentivirus was also used for HSPC imaging of NUP98–KDM5A, NUP98–PRRX1, and NUP98–LNP1. Retrovirus was used for the colony-forming unit assay with NUP98–KDM5A, NUP98–PRRX1, and NUP98–LNP1.

### Colony-Forming Unit Assays

HSPCs isolated and prepared as above were plated in Methocult containing myeloid and erythroid growth factors (M3434, STEMCELL Technologies). Eight thousand cells in 400 μL IMDM (Gibco) containing 2% FBS (HyClone) and 1× penicillin–streptomycin–glutamine (Gibco) were added to 3.2 mL Methocult along with 800 μL of the same media containing 50 ng/mL GM-CSF (PeproTech); 1.1 mL of the Methocult mixture was plated in a 6-cm dish (2,000 cells/dish) in technical triplicates. After 1 week of growth, colonies were counted, and cells were washed well with PBS before being replated as described above. This process was repeated until no colonies were counted across the three plates for each condition or for at least four rounds of colony growth. Images of colonies were acquired using the Nikon Eclipse TS100 microscope. For all NHA9 constructs, colony formation was assessed using mEGFP-tagged constructs. Untagged constructs were used for colony formation assessment of NUP98–KDM5A, NUP98–PRRX1, and NUP98–LNP1.

### PDX Cells

A PDX was established by tail-vein injection of CD3-depleted cells from a female patient with AML FAB M5, karyotype 46XX, and an NUP98–KDM5A fusion into sublethally irradiated (250 rad) NSG-SGM3 mice. Leukemic cells harvested from the abdominal mass were fixed and stained as described above. The patient from whom the PDX cells were obtained was enrolled on a protocol with provision of banking of leukemia samples for research that required written informed consent from the patient or the patient's guardians.

### RNA Isolation

HSPCs were isolated in parallel from 6- to 12-week-old male and female C57Bl/6 mice and transduced with each virus in triplicate. Samples were cultured for 2 days, and mCherry-positive empty vector and mCherry- and mEGFP-positive NHA9 or mutant cells were isolated using FACS. Cells were grown in Methocult for 1 week as described above, and then washed in PBS and resuspended in 600 μL cold TRIzol. Lysis and homogenization were performed immediately using pipetting and vortexing, respectively. RNA was isolated using the Direct-Zol RNA MiniPrep Kit (Zymo Research). RNA was quantified using NanoDrop 2000 (Thermo Fisher Scientific).

### RNA Sequencing and Analysis

RNA-seq was performed by the Hartwell Center at St. Jude Children's Research Hospital. Libraries were prepared using the TruSeq Stranded Total RNA kit (Ilumina). One hundred fifty base-pair, paired reads were generated by sequencing on NovaSeq 6000 (Ilumina). All data have been deposited in the Gene Expression Omnibus (GEO) and are accessible using accession number GSE185621. See Supplementary Information for additional details.

### Confocal Microscopy Imaging

All microscopy images were acquired on a 3i Marianas system configured with a Yokogawa CSU-W spinning disk confocal microscope utilizing a 100× Zeiss objective, 405 nm (Hoechst) and 488 nm (mEGFP) laser lines, and SlideBook 6.0 (3i). 3-D images of cells were captured as z stacks with 0.2 μm spacing between planes, spanning 12.2 μm in total. Live HEK293T cells were imaged at 37°C in phenol red–free DMEM with high glucose (Gibco) supplemented with 1× penicillin/streptomycin, 10% FBS, 6 mmol/L l-glutamine, and 25 mmol/L HEPES. Live mouse HSPCs were imaged in phenol red–free IMDM (Gibco) supplemented with 20% FBS (HyClone), 1× penicillin–streptomycin–glutamine (Gibco), 50 ng/mL SCF (PeproTech), 40 ng/mL Flt3 (PeproTech), 30 ng/mL IL6 (PeproTech), 20 ng/mL IL3 (PeproTech), and 10 ng/mL IL7 (PeproTech). All fixed cell imaging was performed at room temperature.

### Image Analysis of NHA9 Constructs and Additional NUP98 FOs

Analyses of puncta were performed using a customized Python script, in which nuclei were segmented in individual z-layers and then combined into 3-D stacks. Puncta were segmented by filtering the mEGFP channel with a scale-adapted Laplacian of Gaussian (LoG) filter, thresholding the result, and applying watershed segmentation, using the maxima of the LoG-filtered image as seeds. Individual puncta were quantified by computing their volume, mean mEGFP intensity, and integrated mEGFP intensity (the sum of intensities of all puncta pixels). Individual cell nuclei were quantified by computing total and average puncta volume (*V_p_*), mean and integrated mEGFP fluorescence intensity, and mean and integrated puncta localized mEGFP fluorescence intensity. We reported puncta number per unit volume of the cell nuclei because a small proportion of cells were not fully imaged. We quantified the relationship between mEGFP and Hoechst intensities using Pearson correlation. The details of the image analysis pipeline and the extracted puncta features, including partition coefficient (44), are provided in Supplementary Information. Heat maps shown in Fig. 7D were generated using Imaris Imaging Software (Oxford Instruments).

### Data Availability Statement

The RNA-seq data generated in this study have been deposited in GEO and are accessible using the accession number GSE185621. The computer code used to analyze cell imaging data and the primary images are available on Dropbox: https://www.dropbox.com/sh/67c1ppi0m8cud49/AADz4yRIwULNEu85cpSfyvETa?dl=0.

## Supplementary Material

Supplementary Data

Supplementary Data

Supplementary Data

Supplementary Data

Supplementary Data

Supplementary Data

Supplementary Data

Supplementary Table
