# Supplementary material for: Phase Separation Mediates NUP98 Fusion Oncoprotein Leukemic Transformation
Source: Cancer Discov. 2021 Dec 13;12(4):1152–69. doi: 10.1158/2159-8290.CD-21-0674 (PMC8983581; doi:10.1158/2159-8290.CD-21-0674)
Supplement: Supplementary Data [file cd-21-0674_supplementary_data_suppsd1.pdf]

Supplementary Material

Supplemental Methods

Sequences of mEGFP and Fusion protein isoforms and break points

Protein expression plasmids for fluorescence live cell imaging with NUP98 FOs encoded monomeric enhanced green fluorescent protein (mEGFP; residues 1-239 of Accession number AYN72675). Empty vector control plasmids encoded enhanced green fluorescent protein (EGFP; residues 1-239 of Accession number AAG49427). The NUP98-HOXA9 (NHA9) mEGFP-tagged expression construct encodes residues 1-469 of NUP98 (sequence ID: nuclear pore complex protein NUP98-NUP96 isoform 5 [Homo sapiens], Sequence ID: NP_001352054.1) fused to residues 164-272 of HOXA9 (sequence ID: homeobox protein HOXA9 [Homo sapiens], Sequence ID: NP_689952.1). The NUP98-PRRX1 mEGFP-tagged expression construct encodes residues 1-253 followed by residues 263-469 of NUP98 (sequence ID: nuclear pore complex protein NUP98-NUP96 isoform 5 [Homo sapiens], Sequence ID: NP_001352054.1) fused to the residue, D, followed by residues 82-217 of PRRX1 (sequence ID: paired mesoderm homeobox protein 1 isoform PMX-1a [Homo sapiens], Sequence ID: NP_008833.1). The NUP98-KDM5A mEGFP-tagged expression construct encodes residues 1-514 of NUP98 (sequence ID: nuclear pore complex protein NUP98-NUP96 isoform 5 [Homo sapiens], Sequence ID: NP_001352054.1) fused to residues 1485-1690 of KDM5A (sequence ID: lysine-specific demethylase 5A [Homo sapiens], Sequence ID: NP_001036068.1).The NUP98-LNP1 mEGFP-tagged expression construct encodes residues 1-514 of NUP98 (sequence ID: nuclear pore complex protein NUP98-NUP96 isoform 5 [Homo sapiens], Sequence ID: NP_001352054.1) fused to the sequence, N-GQAFSIWHHLY-C, followed by residues 17-213 of LNP1 (sequence ID: hCG2023574 [Homo sapiens], Sequence ID: EAW79821.1).

Plasmid Generation

Plasmids for mammalian cell expression were generated via ligation of synthetic geneblock fragments (IDT) containing Nco1 and Xba1 restriction sites into the CL20 backbone. Sequences were confirmed with whole plasmid sequencing (MGH CCIB DNA Core). Plasmids used to prepare viruses to express NHA9 and mutants in HSPCs were generated in the CCLMPC backbone (supplied by the St. Jude Children's Research Hospital vector core), which uses an MND promoter to drive expression of the gene of interest and a second PGK promoter to drive expression of the reporter gene (mCherry). Plasmids used to prepare viruses to express other NUP98 FOs (NUP98-KDM5A, NUP98-PRRX1, and NUP98-LNP1) for imaging were prepared similarly using the CCLMPC backbone. Plasmids for other NUP98 FOs for colony forming unit assays were generated in a bicistronic retroviral backbone with the MSCV promoter driving expression of the gene of interest. For these other NUP98 FOs, viruses were prepared in HEK293T cells with co-transfection of gagpol and CAG-ECO helper plasmids. Plasmids to express NHA9 and mutants in HEK293T cells were generated in the CL20 backbone which uses a CMV promoter to drive N-terminally mEGFP-tagged NHA9 constructs. Plasmids to express NHA9 and mutants *in vitro* were generated in the pET28 backbone (Novagen). See **Suppl. Table 4** for construct sequences.

Immunophenotyping

After each week of growth in Methocult, remaining cells were cryopreserved in FBS (Hyclone) with 10% DMSO. For immunophenotyping, cells were thawed quickly at 37°C and then washed well in PBS. Mouse HSPC samples were stained for 20-30 minutes in the dark at 4°C with 0.5-1 μL of each of the following antibodies: CD117 (APC-Cy7, Biolegend, RRID:AB_2632808), FCε receptor (APC, Biolegend, RRID:AB_10640121), CD11b (BV650, Biolegend, RRID:AB_2566568), and Gr1 (PE-Cy7, Biolegend, RRID:AB_313381). PDX samples were similarly stained with human CD45 (APC-Cy7, BD Biosciences, RRID:AB_10897014) and human

CD33 (PE-Cy7, BD Biosciences, RRID:AB_399961). Cells were washed again in PBS + 2% FBS (Hyclone) and 1 μL of DAPI staining solution (Miltenyi Biotec) was added immediately prior to analysis on a BD Fortessa flow cytometer. Data were analyzed using FlowJo (v10.7.1).

RNA Sequencing Analysis

Read alignment to the Mus musculus reference genome (mm10) was performed with STAR software (v2.7.0d)[1]. Quality control was performed to identify duplicated and unmapped reads; all samples were of acceptable quality for analysis. To evaluate gene expression level, the read count for each annotated gene was calculated using HTSeq[2] (version 0.11.2). Differential gene expression and regularized log transformation (rlog) on raw count data were carried out using DESeq2[3]. All the genes were ranked according to the fold-change and significance from differential analysis. Gene set enrichment analysis was then performed using molecular signatures database (MSigDB) (version 6.2) C2 genes[4].

Cell fixation and immunofluorescence

To fix cells for nuclear pore complex staining, 250,000 cells were grown on Poly-L-Lysine-treated (Sigma-Aldrich), sterile coverslips (VWR). HEK293T cells were first rinsed with warm 1X PBS, followed by a 20 min incubation with 4% Paraformaldehyde (Electron Microscopy Sciences), then a 5-minute incubation in 0.5% Triton-X-PBS for cell permeabilization. Cells were washed with Wash Buffer (0.1% Triton-X-PBS) and blocking was performed at room temperature (RT) for 1 hour in Blocking Buffer [10% Normal Donkey Serum (Jackson ImmunoResearch)] prior to primary antibody incubation. For fixation of hematopoietic cells and NUP98-KDM5A PDX cells, a cytocentrifuge was used to adhere cells to a glass slide by spinning at 400 rpm for 4 minutes. The cells were then rapidly rinsed in 1X PBS-5mM EGTA, followed by incubation at -20 °C in 95% Methanol-5 mM EGTA for 30 minutes. Cells were washed with 1X PBS, followed by blocking at room temperature for 1 hour prior to primary antibody incubation. All primary antibodies were

diluted in 5% Normal Donkey Serum and added to coverslips overnight at 4 °C. The primary antibodies used were Mouse anti-NUP107 (Abcam, Mab414, RRID:AB_448181, 1:300) and Rat anti-NUP98 (GeneTex, 2H10, RRID: AB_2894964, 1:200). After primary antibody incubation, cells were washed and incubated for 45 minutes at RT with secondary antibodies conjugated to Alexa Fluor Rhodamine Red™-X or Alexa Fluor 647 (Jackson ImmunoResearch; RRID:AB_2340614) at 1:300 diluted in 5% Normal Donkey Serum. Cells were washed and counter stained with DAPI (4',6-Diamidino-2-Phenylindole, Dihydrochloride, Invitrogen) diluted in PBS (300 nM) for 2 minutes, and then mounted onto glass slides with antifade solution (90% glycereol, 0.5% N-propyl gallate).

Gel mobility shift assays

The DNA-binding assays for HOXA9 homeodomain ($HOXA9_{197\text{-}272}$ HD) and with DNA binding-deficient mutant ($HOXA9_{197\text{-}272}$-ΔDNA; mutations include R258A, K262A, and W199G) were performed using the concentration range from 2.5 nM to 1.25 μM protein. The DNA concentration was kept constant at 10 nM. A double-stranded 20 base pair DNA oligonucleotide (the sequences 5′-ACTCTATGATTTACGACGCT-3′; HOXA9 binding site, TTTAC) having a 5′ end with Cy5 dye (Integrated DNA Technologies, Inc., USA) was chosen for the assay. Both the protein and DNA were present in 10 mM Tris (pH 7.5), 75 mM NaCl, 6% glycerol, 1 mM DTT, 1 mM EDTA, 6.7 ng/μL Poly(2′-deoxyinosinic-2′-deoxycytidylic acid) sodium salt (dIdC), 6.7 ng/μL BSA. After mixing the DNA and protein, the reactions were incubated at room temperature for 30 min and then run on a 16% acrylamide gel in 1x TBE (Invitrogen) buffer at a constant voltage of 100 V for 140 min. The gel was imaged in a gel imager (Amersham Imager 600, GE Healthcare Life Sciences, USA) for Cy5 fluorescence.

Recombinant protein expression, purification, and fluorescent labeling

Cultures transfected with pET28a-based plasmids expressing the NHA9 and other protein constructs (see Suppl. Table 3) were grown at 37 °C to an optical density at 600 nm (OD600) ~ 0.8. Protein expression was induced by the addition of 1 mM IPTG (GoldBio) and the cultures were incubated at 37 °C for 4 h. 8 L of bacterial culture were harvested by centrifugation and lysed in Buffer A (20 mM Tris, 500 mM NaCl, 1 mM Tris (2-carboxyethyl) phosphine hydrochloride (TCEP), pH 7.4) containing 0.1 % Triton X-100, by sonication on ice. The cell lysate obtained was centrifuged at 30,000 x g at 4 °C for 30 minutes. The supernatant was discarded except in the cases of $NHA9_{Midi}$-21FGAA, $HOXA9_{197-272}$ HD and $HOXA9_{197-272}$ HD-ΔDNA which are found in the soluble fraction. For all other proteins, the inclusion bodies obtained were dissolved in extraction buffer [6 M guanidine HCl (GdnHCl) in Buffer A]. The solution was again sonicated and centrifuged. The supernatant obtained after centrifugation of the GdnHCl/Buffer A solutions and the soluble fractions of $NHA9_{Midi}$-21FGAA, $HOXA9_{197-272}$ HD and $HOXA9_{197-272}$ HD-ΔDNA were loaded onto a Ni-NTA column (30 mL). The bound proteins were washed with two column volumes of Wash Buffer (6 M urea in Buffer A) followed by 5 column volumes of Wash Buffer containing 50 mM Imidazole. The proteins were eluted with 6 M urea in Buffer A containing 500 mM Imidazole. The 12x His tag was cleaved in an overnight dialysis step at room temperature, against 20 mM Tris pH 7.4, 150 mM NaCl, 2 M urea, 1 mM TCEP buffer, in the presence of TEV protease. For $NHA9_{Midi}$-21FGAA, $HOXA9_{197-272}$ HD and $HOXA9_{197-272}$ HD-ΔDNA, the TEV digestion was carried out in PBS buffer (1x PBS; 136.9 mM NaCl, 2.68 mM KCl, 10 mM Na_2HPO_4 1.7 mM KCl, pH 7.4) at 4 °C overnight. The 12x His tagged TEV was then removed using Ni-NTA column and the flow-through fractions containing cleaved proteins were loaded onto a HPLC column (PLRP-S 1000A 8 μM, Agilent Technologies) equilibrated with Mobile Phase A (0.1 % TFA and 5 % acetonitrile in water) and eluted with a linear gradient of Mobile Phase B (0.1% TFA in acetonitrile). The fractions with high purity were identified by SDS-PAGE, flash frozen and lyophilized. For labeling NHA9, a single cysteine construct (C188A) was created. All proteins containing a single cysteine (excluding NHA9-21FGAA, NHA9-8FA and NUP98-N) were fluorescently labeled using

maleimide derivatives of Alexa Fluor dyes (Thermo Fisher Scientific), according to the manufacturer's protocol. The lyophilized proteins (400-500 µM) were mixed with Alexa Fluor dye at 1:3 ratio in 6 M guanidine HCl (GdnHCl), 20 mM Tris, 5 mM EDTA, pH 7.0, containing 1 mM Tris (2-carboxyethyl) phosphine hydrochloride (TCEP). After overnight incubation, the reaction was quenched with 50mM DTT. The NHA9-21FGAA, NHA9-8FA and NUP98-N proteins were labeled at the N-terminus using the NHS ester of Alexa Fluor dyes (Thermo Fisher) at a 1:4 (protein:Alexa Fluor dye) mole ratio in 1 M GdnHCl, 20 mM HEPES, pH 7.2, containing 1 mM TCEP. To quench the reaction, 50 mM of Tris was added. All of the labeled proteins were passed purified using HPLC (PLRP-S 1000A 8 μM, Agilent Technologies) to remove any unreacted/free dye. For imaging samples between 315 nM to 20 μM [protein], 100 nM Alexa Fluor-labeled NHA9 constructs were used; however, for concentrations between 10 nM to 160 nM, a final concentration of 10 nM Alexa Fluor-labeled of the NHA9 constructs was used.

Sequence analysis

We used a custom, Matlab-based computational pipeline called Swiss Army Knife (SAK) to analyze the amino acid sequence features of NHA9. The SAK pipeline identifies and annotates low-complexity regions by calculating the sequence Shannon Entropy[5,6] at each position, conserved domains and features by accessing the Conserved Domain Databases (CDD)[7-9], predicted secondary structure using the JPred4 API[10], predicted disordered regions and Molecular recognition features (MoRFs) by executing IUPred2A[11], regions predicted to be enriched in Pi-Pi interactions and predicted prion-like domains using PScore[12] and PLAAC[13], respectively, acidic and basic tracts in disordered regions using ABTScore[14], hydropathy utilizing the Kyte-Doolittle hydropathy scale[15] as implemented in CIDER[16], position-specific amino acid identities, and enrichment of amino acids for the overall sequence as compared to frequencies in the human proteome as reported in UniProtKB release 15[17]. The results of this analysis are then

exported as graphical summaries showing site-specific sequence features, as shown in Suppl. Figs. 2I and 8.

Confocal fluorescence microscopy image analysis of NHA9 constructs and additional NUP98 FOs

For all quantified images, the x and y dimensions were 0.11 μm × 0.11 μm, with 992 total pixels in each dimension. The z-step size was 0.20 μm, and 61 planes were image, giving 61 pixels in the z dimension. The volume for each 3D image is 61 × 992 × 992 pixels. Therefore, one cubic pixel volume (voxel) corresponds to (0.11 μm × 0.11 μm × 0.20 μm) = 0.00242 μm^3. Individual puncta volume was determined in the unit of cubic pixel and converted to μm^3 using the abovementioned conversion.

Background intensity correction

For each 3D image stack, background intensity correction was performed for each pixel based on the peak background intensity value at 101 a.u. in the mEGFP channel of untransfected images (see Suppl. Fig. 7K). For background correction, this background fluorescence value was subtracted from the fluorescence intensity of all image pixels. All the 3D images were used for image analysis after background correction, as described below.

Cell nuclei segmentation

Cell nuclei were segmented based on the DNA (Hoechst) channel using Cellpose[18] (v 0.6.5). Since each 3D dataset represented only one layer of cells, cell nuclei were segmented in 2D in each z-layer and then combined into 3D stacks. Each individual z-layer intensity was normalized between the 0.25^{th} and 99.75^{th} percentiles and segmented with parameters: model type:" cyto",

diameter: 120 pixels (13.2 µm), flow threshold: 0.4, probability threshold: 0. The cytoplasm model performed better than the nucleus model for our datasets.

After segmenting nuclei in individual z-layers, results were combined into 3D stacks as follows: The total area of segmented nuclei was computed in each z-layer (excluding 10 layers at each border), and the layer with maximal total area was used as a reference for cell IDs. Cellpose segmentations of individual z-layers were then combined into a 3D stack, converted to a binary mask, and filtered with a 3D median filter (size 3 pixels), to remove objects that were detected in only one z-layer and fill gaps between masks that were one-layer wide. Finally, the binary mask of each z-layer was multiplied by the labels of the reference layer to assign cell IDs throughout the entire 3D stack.

Puncta segmentation

Detection and segmentation of puncta in the mEGFP channel were based on applying the scale-adapted Laplacian of Gaussian (LoG) filter[19]. We first detected puncta centers using the blob_log (LoG detector) function of the scikit-image library[20] with parameters: min_sigma=[1, 1.8, 1.8] (0.2 µm), max_sigma=[10, 18.2, 18.2] (2 µm), num_sigma=5, overlap=1, threshold=0.003 for the NHA9 constructs and NUP98 FOs images in HEK293T cells (threshold=0.001 for the NHA9 constructs and NUP98 FOs images in HSPC cells, and NUP98-KDM5A patient derived xenograft (PDX) and the control human CD34+ cells). Puncta centers were additionally filtered according to intensity relative to the background mEGFP signal. To quantify the background mEGFP signal in each image, we computed a median mEGFP intensity in each cell, and then calculated the 95th percentile of these values over the entire image. Puncta centers with an intensity lower than 3 times the background mEGFP signal were removed for the NHA9 constructs and NUP98 FOs images in HEK293T cells. The same was done for the NUP98 signal in quantifying the NUP98-KDM5A PDX and the control human CD34+ cells. For the NHA9 constructs and NUP98 FOs

images in HSPC cells puncta centers with an intensity < 2 times the background mEGFP signal were removed. For quantifying puncta detected by the NUP98 antibody in NUP98-KDM5A PDX and control human CD34+ cells (**Suppl. Fig. 7N, O**), all puncta within 0.5 μm of the nuclear periphery were excluded to avoid fluorescence signal of endogenous NUP98 at the nuclear pore complex.

To segment individual puncta, we filtered the image with five LoG filters with scales linearly interpolated between min_sigma and max_sigma using the filters.gaussian and filters.laplace functions of scikit-image (v 0.18.1). We then calculated the pixel-wise maximum of filter responses. The result was thresholded at 0.001 (relative intensity in LoG scale units) for the NHA9 constructs and NUP98 FOs images in HEK293T cells and processed with a distance transform watershed using the puncta centers as seeds. For the NHA9 constructs and NUP98 FOs images in HSPC cells, and NUP98-KDM5A PDX and the control human CD34+ cells, the threshold for puncta segmentation was 0.0005.

Conversion of mEGFP fluorescence intensity to protein concentration

We converted the total mEGFP fluorescence intensity per pixel volume to the GFP molar concentration based on the calibration plot shown in **Suppl. Fig. 1A.** This was done for the following parameters: [G-NHA9 construct], [LP], and [DP], as reported in the main figures and supplementary figures. For this calibration, 22 mEGFP protein solutions were prepared as well as a buffer-only solution (without mEGFP), with concentrations of mEGFP ranging from 1 nM to 100 μM. For each solution, 61 z-stack (0.2 μm step size ranging 12.2 μm total) images were recorded at 6 different positions using a single fluorescence channel for mEGFP. We used the image analysis pipeline discussed above to determine the total mEGFP fluorescence intensity from the images. We computed that one absorbance unit of total mEGFP fluorescence intensity per cubic pixel volume corresponds to a mEGFP concentration of 20 nM (**Suppl. Fig. 1A**). We

used this conversion factor to convert mEGFP fluorescence intensities per unit volume into mEGFP molar concentrations, i) for each cell nucleus ([G-NHA9 construct]), ii) for the light phase of each nucleus ([LP]) and within puncta within each nucleus, determined as the average over all detected puncta ([DP]). We used the same conversion method for the images of G-NUP98 FOs in HEK293T cells and G-NHA9 constructs in HSPC cells, as well.

Extraction of thermodynamic features from image analysis

We applied several selection criteria during the analysis of thermodynamic parameters based upon the confocal fluorescence microscopy images of the NHA9 constructs, as follows. We excluded the cells based on the following conditions, (i) cells with a [G-NHA9 construct] value below ~0.02 μM, determined as the background level of fluorescence in the mEGFP channel of untransfected HEK293T cells (**Suppl. Fig. 1B**) and (ii) incomplete cells, determined as cells containing puncta near the periphery of the images using 5-pixel cutoff in the xy plane. We used these cell selection criteria for the images of G-NUP98 FOs in HEK293T cells and G-NHA9 constructs in HSPC cells.

We computed the average mEGFP concentration within puncta (dense phase) per cell,

$$[DP] = \left(\sum_{i=1}^{n} \frac{Puncta\ GFP\ FL\ intensity}{Puncta\ volume}\right)/n$$

where *n* is the number puncta per cell and the volume of each punctum is in the unit of cubic pixels. We calculated the average mEGFP concentration in the non-puncta containing region of the nucleoplasm (light phase) per cell,

$$[LP] = \frac{Total\ nuclear\ GFP\ FL\ intensity\ -\ Total\ puncta\ GFP\ FL\ intensity}{Nucelar\ volume\ -\ Total\ puncta\ volume}$$

where the nuclear and total puncta volume are in the unit of cubic pixels.

From the quantification of the individual cell nuclei and individual puncta, we obtained the average concentration of the NHA9 constructs / NUP98 FOs in the nucleoplasm (defined as, light phase) per cell ([LP]) and average concentration within puncta (defined as, dense phase) per cell ([DP]). The ratio of the concentration of the dense to the light phase is defined as the partition coefficient [21], $K_p = \frac{[DP]}{[LP]}$. The Gibbs free energy of transfer (into puncta) is defined as, ΔG_{Tr} = -RT *ln*(K_p), where R is the gas constant (= 1.98×10^{-3} kcal/ mol/ K) and T is temperature (= 310 K).

Quantification of nuclear peripheral puncta in HEK293T and mHSPCs

We quantified the nuclear peripheral puncta using distance transform method by calculating the Euclidean distance to the nucleus border for each pixel of the nucleus mask, and then averaged those values for individual puncta. This way we estimated the average distance of all puncta pixels to the nucleus border (per puncta). To obtain the percentage of nuclear peripheral puncta for each construct, we identified the number of puncta within a distance $\leq$ 0.5 μm from the nuclear periphery and compared that with the total number of puncta.

Statistical analysis of the image quantification data

Generalized linear mixed effects models[22] were used to account for the random effects of image position while comparing cellular imaging characteristics across two constructs. A Poisson model with log link was used for analysis of puncta counts. A Gaussian model with identity link was used for analysis of all other imaging characteristics. Puncta count and the transfer free energy (ΔG_{Tr})

(see the main text Methods) were not transformed prior to analysis. All other cellular imaging variables were $\log_{10}$-transformed prior to analysis to be more accurately modeled by a normal distribution. All p-values are two-sided. No multiple testing adjustments were performed. All the pairwise p-values were calculated using R-package, lme4 (v 1.1-26) and lmerTest (3.1-3).

All the plots of puncta and thermodynamic features were performed using R-package (v 4.1.0), ggscatter from ggpubr (v 0.4.0) and ggplot2 (v 3.3.5).

References

1 Dobin, A. *et al.* STAR: ultrafast universal RNA-seq aligner. *Bioinformatics* **29**, 15-21, doi:10.1093/bioinformatics/bts635 (2013).

2 Anders, S., Pyl, P. T. & Huber, W. HTSeq--a Python framework to work with high-throughput sequencing data. *Bioinformatics* **31**, 166-169, doi:10.1093/bioinformatics/btu638 (2015).

3 Love, M. I., Huber, W. & Anders, S. Moderated estimation of fold change and dispersion for RNA-seq data with DESeq2. *Genome Biol* **15**, 550, doi:10.1186/s13059-014-0550-8 (2014).

4 Subramanian, A. *et al.* Gene set enrichment analysis: a knowledge-based approach for interpreting genome-wide expression profiles. *Proc Natl Acad Sci U S A* **102**, 15545-15550, doi:10.1073/pnas.0506580102 (2005).

5 Shannon, C. E. The mathematical theory of communication. 1963. *MD Comput* **14**, 306-317 (1997).

6 Adami, C. Information theory in molecular biology. *Physics of Life Reviews* **1**, 3-22 (2004).

7 Marchler-Bauer, A. & Bryant, S. H. CD-Search: protein domain annotations on the fly. *Nucleic Acids Res* **32**, W327-331, doi:10.1093/nar/gkh454 (2004).

8 Marchler-Bauer, A. *et al.* CDD: a Conserved Domain Database for the functional annotation of proteins. *Nucleic Acids Res* **39**, D225-229, doi:10.1093/nar/gkq1189 (2011).

9 Lu, S. *et al.* CDD/SPARCLE: the conserved domain database in 2020. *Nucleic Acids Res* **48**, D265-D268, doi:10.1093/nar/gkz991 (2020).

10 Drozdetskiy, A., Cole, C., Procter, J. & Barton, G. J. JPred4: a protein secondary structure prediction server. *Nucleic Acids Res* **43**, W389-394, doi:10.1093/nar/gkv332 (2015).

11 Meszaros, B., Erdos, G. & Dosztanyi, Z. IUPred2A: context-dependent prediction of protein disorder as a function of redox state and protein binding. *Nucleic Acids Res* **46**, W329-W337, doi:10.1093/nar/gky384 (2018).

12 Vernon, R. M. *et al.* Pi-Pi contacts are an overlooked protein feature relevant to phase separation. *Elife* **7**, doi:ARTN e3148610.7554/eLife.31486 (2018).

13 Lancaster, A. K., Nutter-Upham, A., Lindquist, S. & King, O. D. PLAAC: a web and command-line application to identify proteins with prion-like amino acid composition. *Bioinformatics* **30**, 2501-2502, doi:10.1093/bioinformatics/btu310 (2014).

14 Somjee, R., Mitrea, D. M. & Kriwacki, R. W. Exploring Relationships between the Density of Charged Tracts within Disordered Regions and Phase Separation. *Pac Symp Biocomput* **25**, 207-218 (2020).

15 Kyte, J. & Doolittle, R. F. A simple method for displaying the hydropathic character of a protein. *J Mol Biol* **157**, 105-132, doi:10.1016/0022-2836(82)90515-0 (1982).

16 Holehouse, A. S., Das, R. K., Ahad, J. N., Richardson, M. O. & Pappu, R. V. CIDER: Resources to Analyze Sequence-Ensemble Relationships of Intrinsically Disordered Proteins. *Biophys J* **112**, 16-21, doi:10.1016/j.bpj.2016.11.3200 (2017).

17 UniProt, C. UniProt: the universal protein knowledgebase in 2021. *Nucleic Acids Res* **49**, D480-D489, doi:10.1093/nar/gkaa1100 (2021).

18 Stringer, C., Wang, T., Michaelos, M. & Pachitariu, M. Cellpose: a generalist algorithm for cellular segmentation. *Nat Methods* **18**, 100-106, doi:10.1038/s41592-020-01018-x (2021).

19 Lindeberg, T. Feature Detection with Automatic Scale Selection. *International journal of computer vision* **30**, 79-116 (1998).

20 van der Walt, S. *et al.* scikit-image: image processing in Python. *PeerJ* **2**, e453, doi:10.7717/peerj.453 (2014).

21 Riback, J. A. *et al.* Composition-dependent thermodynamics of intracellular phase separation. *Nature* **581**, 209-214, doi:10.1038/s41586-020-2256-2 (2020).

22 Bates, D., Mächler, M., Bolker, B. & Walker, S. Fitting linear mixed-effects models using lme4. *arXiv preprint arXiv:1406.5823* (2014).

Supplementary video legends

Suppl. Video 1. Time-lapse confocal microscopy video of G-NHA9 puncta in HEK293T cells acquired at an experimental frame rate of **Left,** 113 milliseconds/frame (Video frame rate is 7 frames/second. Time format is mm:ss.), **Right,** 10 seconds/frame (Video frame rate is 1 frame/second. Time format is mm:ss.)

Suppl. Video 2. Time-lapse confocal microscopy video of a fusion event in a HEK293T cell expressing G-NHA9-ΔDNA acquired at an experimental frame rate of 172 milliseconds/frame. Video frame rate is 5.8 frames/second. Time format is mm:ss.

Suppl. Video 3. Time-lapse confocal microscopy video of a fusion event in a HEK293T cell expressing G-NHA9-ΔDNA acquired at an experimental frame rate of 10 seconds. Video frame rate is 5 frames/second. The video is presented as a maximum intensity projection of 10 confocal planes offset by 0.3 μm per plane. Time format is mm:ss.

Suppl. Video 4. Time-lapse confocal microscopy video of a fusion event in a HEK293T cell expressing G-NUP98-N acquired at an experimental frame rate of 5 minutes/frame. Video frame rate is 2 frames/second. Time format is hh:mm.

Suppl. Video 5. Time-lapse confocal microscopy video of G-NHA9 puncta in a lin- HSPC acquired at an experimental frame rate of 1 second. Video frame rate is 7 frames/second. Time format is mm:ss.

Suppl. Videos 6. Time-lapse confocal microscopy video of a fusion event in a HEK293T cell expressing G-NUP98-LNP1 acquired at an experimental frame rate of 1.2 seconds/frame. Video frame rate is 7 frames/second. Time format is mm:ss.

Suppl. Figure 1:

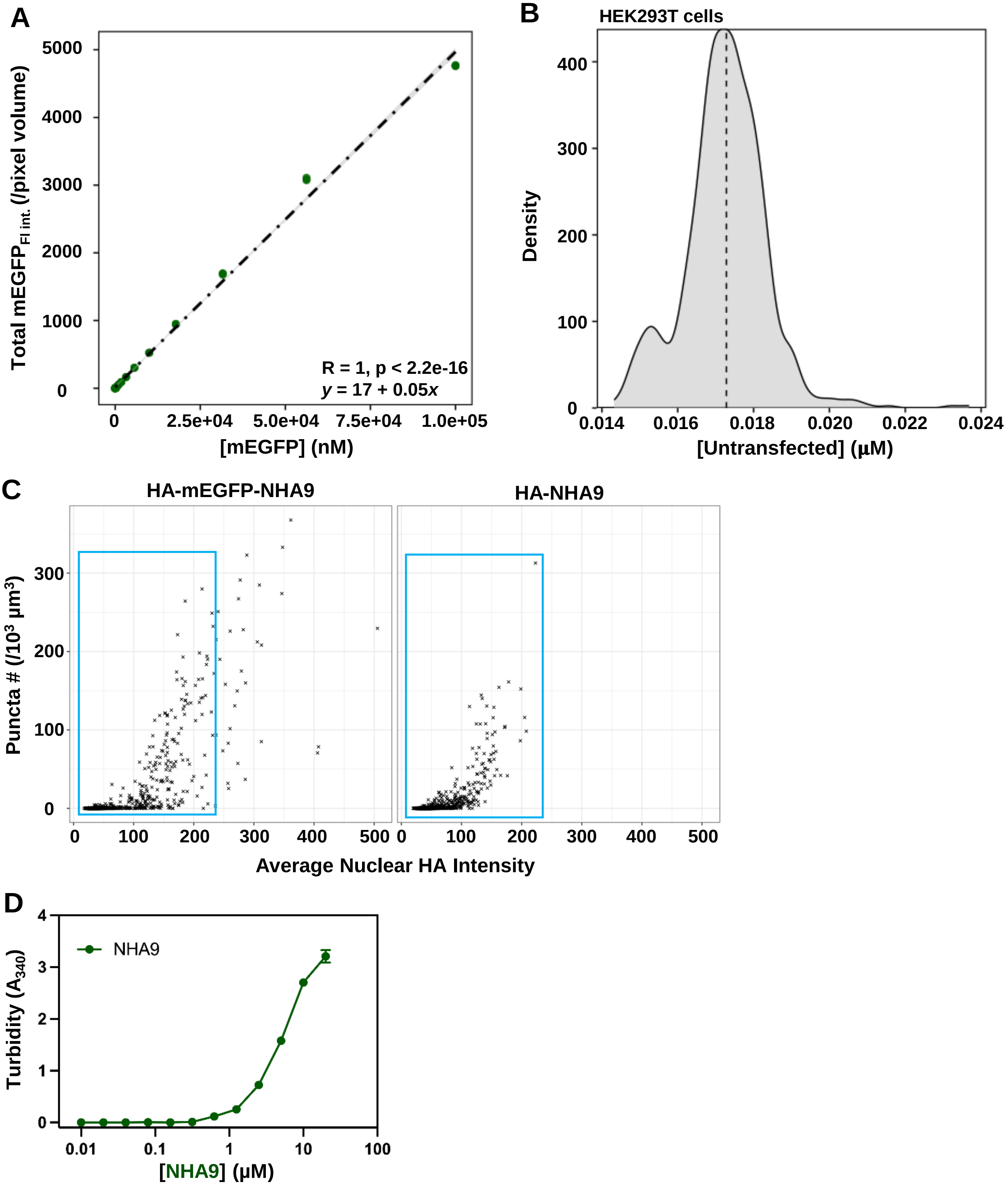

Suppl. Figure 1. A, Calibration of the total mEGFP fluorescence intensity *vs*. mEGFP concentration. From the slope based on linear fitting, we determined that one-unit of mEGFP intensity per pixel volume corresponds to 20 nM. The volume of each image is 60,027,904 (pixels)^3 (992 × 992 × 61). **B**, Density plot of mEGFP concentration from untransfected HEK293T cells used to determine the fluorescence background in the mEGFP channel of the microscope; this was used to establish a fluorescence intensity threshold (in μM mEGFP units) below which cells were rejected for analysis; the median value of 0.02 μM mEGFP was used as the threshold value for analysis of microscopy images for all G-NHA9 constructs and the empty EGFP vector control. **C,** Quantification of nuclear puncta detected by the HA antibody in the presence and absence of a mEGFP tag. In the puncta # (/10^3 μM^3) vs. average nuclear HA intensity plot, the boxed regions highlight equivalent fluorescence intensity ranges for each condition; n = 750 for HA-mEGFP-NHA9 and 760 for HA-NHA9. **D**, Concentration-dependent turbidity values for NHA9 protein monitored by UV absorbance at 340 nm.

Suppl. Figure 2:

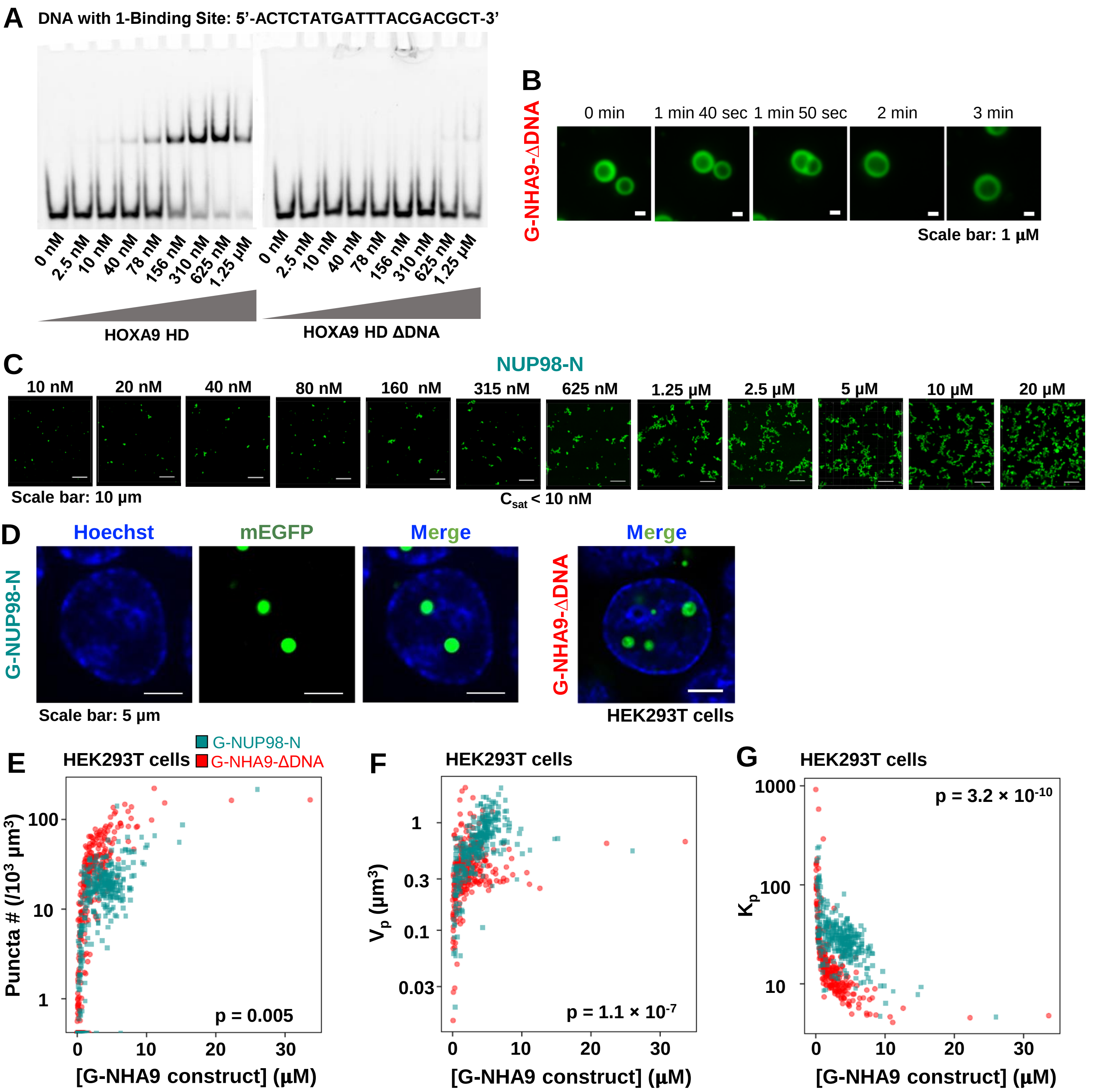

Suppl. Figure 2. Analysis of the effects of mutations in the HOXA9 homeodomain on sequence-specific DNA binding activity. A, Results of gel mobility shift assays with a 20 base pair double-stranded DNA oligonucleotide and the HOXA9 homeodomain ($HOXA9_{197\text{-}272}$ HD) and with DNA binding-deficient mutant ($HOXA9_{197\text{-}272}$-ΔDNA; mutations include R258A, K262A, and W199G). **B**, Still images of multiple time-points from a confocal fluorescence microscopy time-lapse video (**Suppl. Video 3**) for a fusion event in a HEK293T cell expressing G-NHA9-ΔDNA. **C**, Confocal fluorescence micrographs of Alexa 488-labeled NUP98-N condensates prepared *in vitro* with increasing protein concentration. The micrographs are presented as maximum intensity projections of 13 confocal planes offset by 0.5 μm per plane. **D,** Representative confocal microscopy images of live HEK293T cells expressing G-NUP98-N (green). DNA is stained with Hoechst dye (blue). The merge image of G-NHA9-ΔDNA puncta with stained DNA is included for comparison. **E-G,** Plots of puncta # (/10^3 μm^3) (**E**), V_p (μm^3) (**F**), and K_p ($K_p = \frac{[DP]}{[LP]}$) (**G**), *vs.* [G-NHA9 construct] for G-NUP98-N (teal) and G-NHA9-ΔDNA (red). Data is plotted on a semi-log (y-axis: $\log_{10}$) scale. The pairwise p-value between G-NUP98-N *vs.* G-NHA9-ΔDNA is shown in each plot (**E-G**) (n = 846 and 780 in (**E**) including the cells with zero punctum and n = 318 and 254 in (**F-G**) excluding the cells with zero punctum, respectively, for G-NUP98-N *vs.* G-NHA9-ΔDNA).

Suppl. Figure 2 (Cont.):

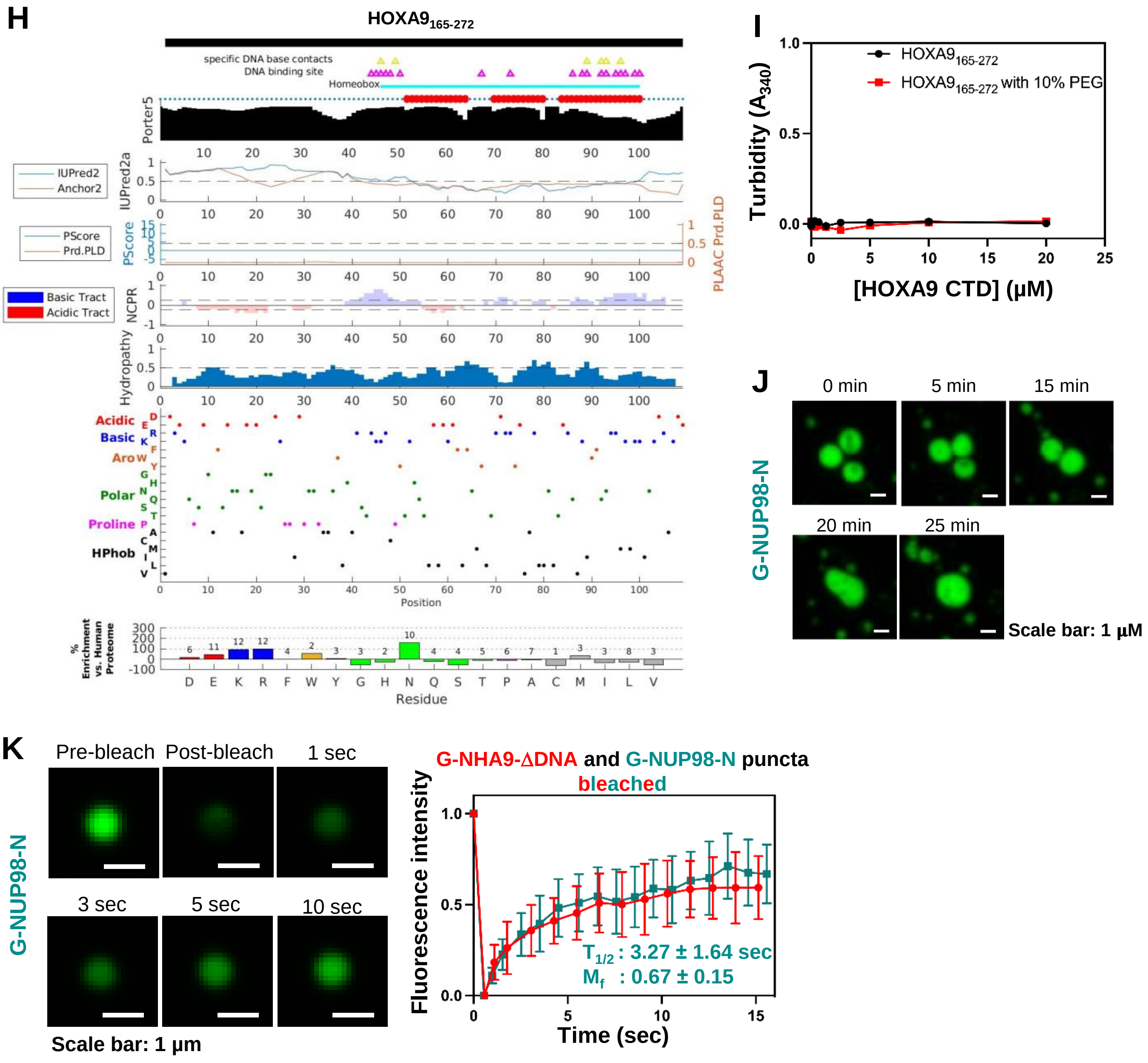

Suppl. Figure 2 (cont.). H, Results of the sequence analysis pipeline show sequence features, including Shannon entropy, predicted secondary structure, predicted disorder, presence of cation-pi and pi-pi interactions, prion-like domains, acidic and basic tracts, and the occurrence and enrichment of amino acids within the HOXA9 region of NHA9. **I**, Concentration-dependent turbidity values for the C-terminal HOXA9 region of NHA9 in the presence (red) and absence (black) of 10% PEG monitored by UV absorbance at 340 nm. **J**, Still images of multiple time-points from a confocal fluorescence microscopy time-lapse video **(Suppl. Video 4)** for a fusion event in a HEK293T cell expressing G-NUP98-N. **K**, Confocal micrographs of fluorescence recovery of a single G-NUP98-N punctum in HEK293T cells at different times after photo-bleaching. FRAP recovery curve (right) for a photo-bleached NUP98-N punctum (teal) with the recovery curve for G-NHA9-ΔDNA (red) included for comparison. Individual puncta were manually tracked at different times and recovery was plotted as the mean ± the standard deviation (S.D.; n = 20).

Suppl. Figure 3:

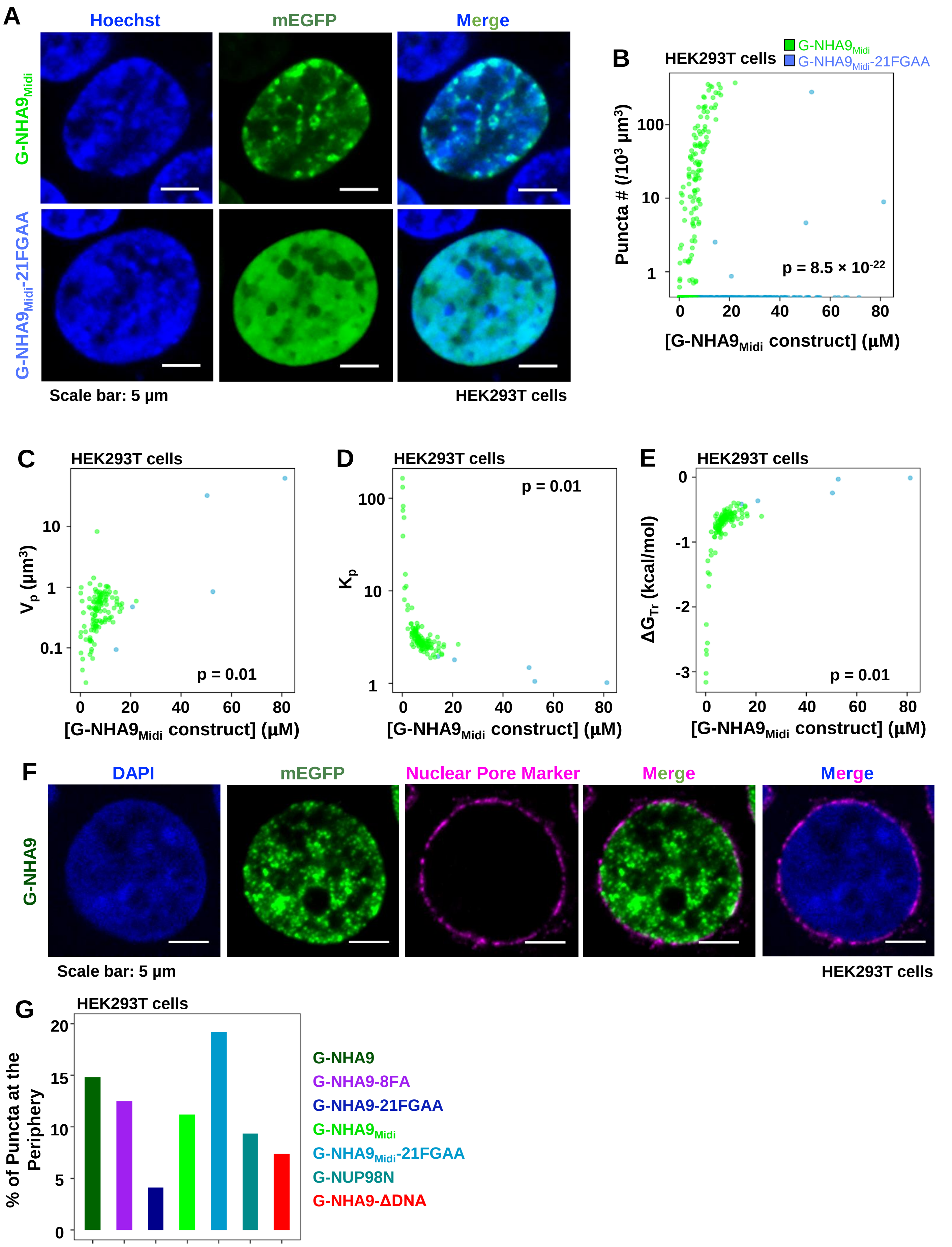

Suppl. Figure 3. Mutation of multiple FG residues in the FG-rich IDR of NHA9$_{Midi}$ disrupts puncta formation in cells. **A,** Representative images of live HEK293T cells expressing G-NHA9$_{Midi}$ (top, green) and G-NHA9$_{Midi}$-21FGAA (bottom, green). DNA is stained with Hoechst dye (blue). **B-E,** Plots of puncta # (/10^3 μm^3) (**B**), V_p (μm^3) (**C**), K_p ($K_p = \frac{[DP]}{[LP]}$) (**D**), and ΔG_{Tr} (kcal/mol) (**E**) vs. [G-NHA9$_{Midi}$ construct] for G-NHA9$_{Midi}$ (lime green) and G-NHA9$_{Midi}$-21FGAA (light blue). Data is plotted on a semi-log (y-axis: $\log_{10}$) scale in (**B-D**). The pairwise p-value between G-NHA9$_{Midi}$ *vs.* G-NHA9$_{Midi}$-21FGAA is shown in each plot (**B-E**) (n = 731 and 791 in (**B**) including the cells with zero punctum, and n = 122 and 5 in (**C-E**) including the cells with zero punctum, respectively, for G-NHA9$_{Midi}$ and G-NHA9$_{Midi}$-21FGAA). **F,** Representative images of fixed HEK293T cells expressing G-NHA9 and stained with a nuclear pore complex marker (NUP107, magenta). DNA is labeled with DAPI dye (blue). **G,** Quantitation of percent of total puncta localized within 0.5 μm of the nuclear periphery for all G-NHA9 constructs in HEK293T cells.

Suppl. Figure 4:

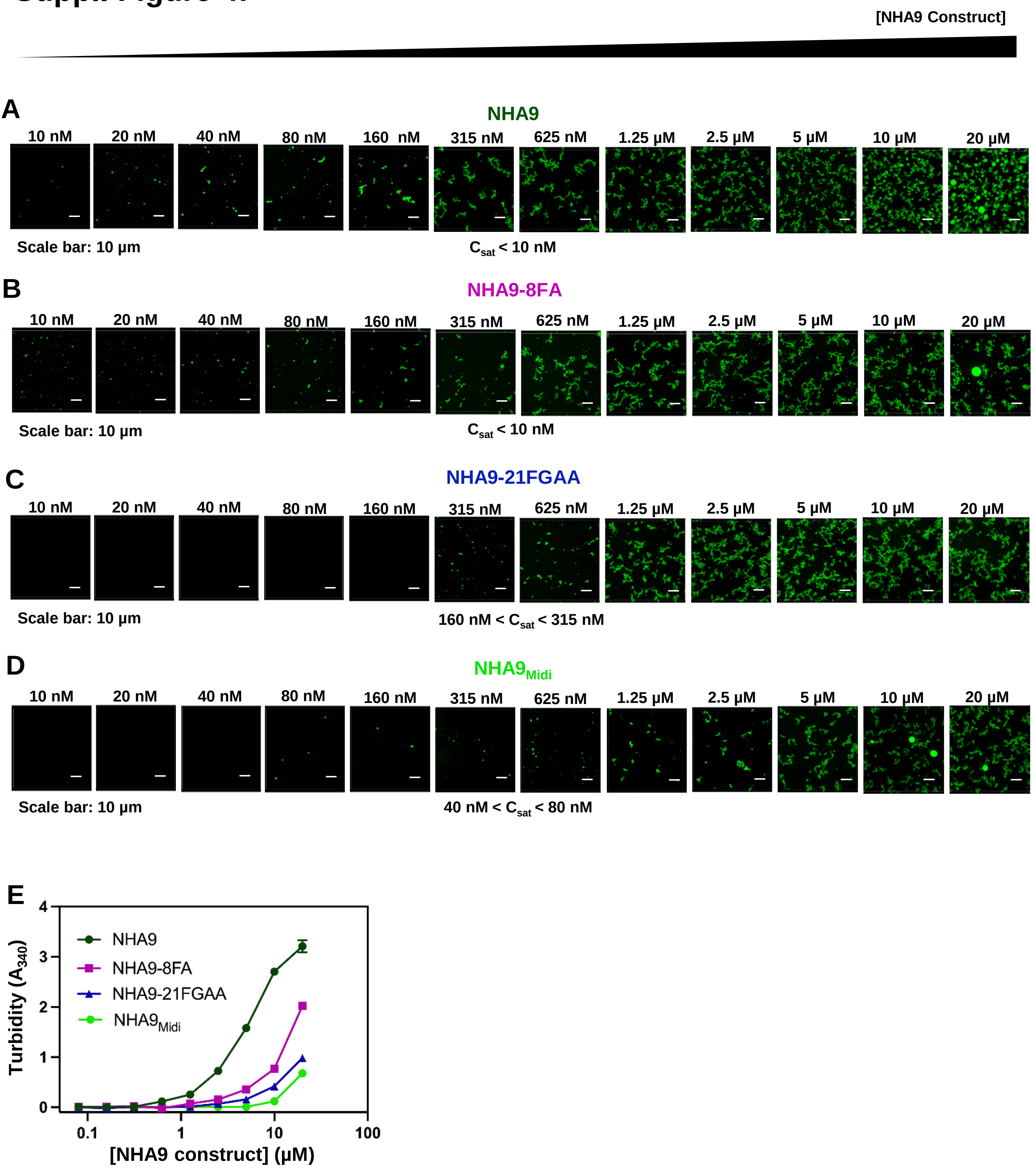

Suppl. Figure 4. ***In vitro* characterization of FG mutant constructs. A-D,** Confocal fluorescence micrographs of Alexa 488-labeled NHA9 (**A**), NHA9-8FA (**B**), NHA9-21FGAA (**C**), and $NHA9_{Midi}$ (**D**) condensates prepared *in vitro* at concentrations from 10 nM to 20 µM. The micrographs are presented as maximum intensity projections of 13 Z-stack images acquired over 6 mm with 0.5 μm resolution. **E,** Concentration-dependent turbidity values of purified NHA9 (green), NHA9-8FA (purple), NHA9-21FGAA (blue), and $NHA9_{Midi}$ (lime green) protein monitored by UV absorbance at 340 nm.

Suppl. Figure 5:

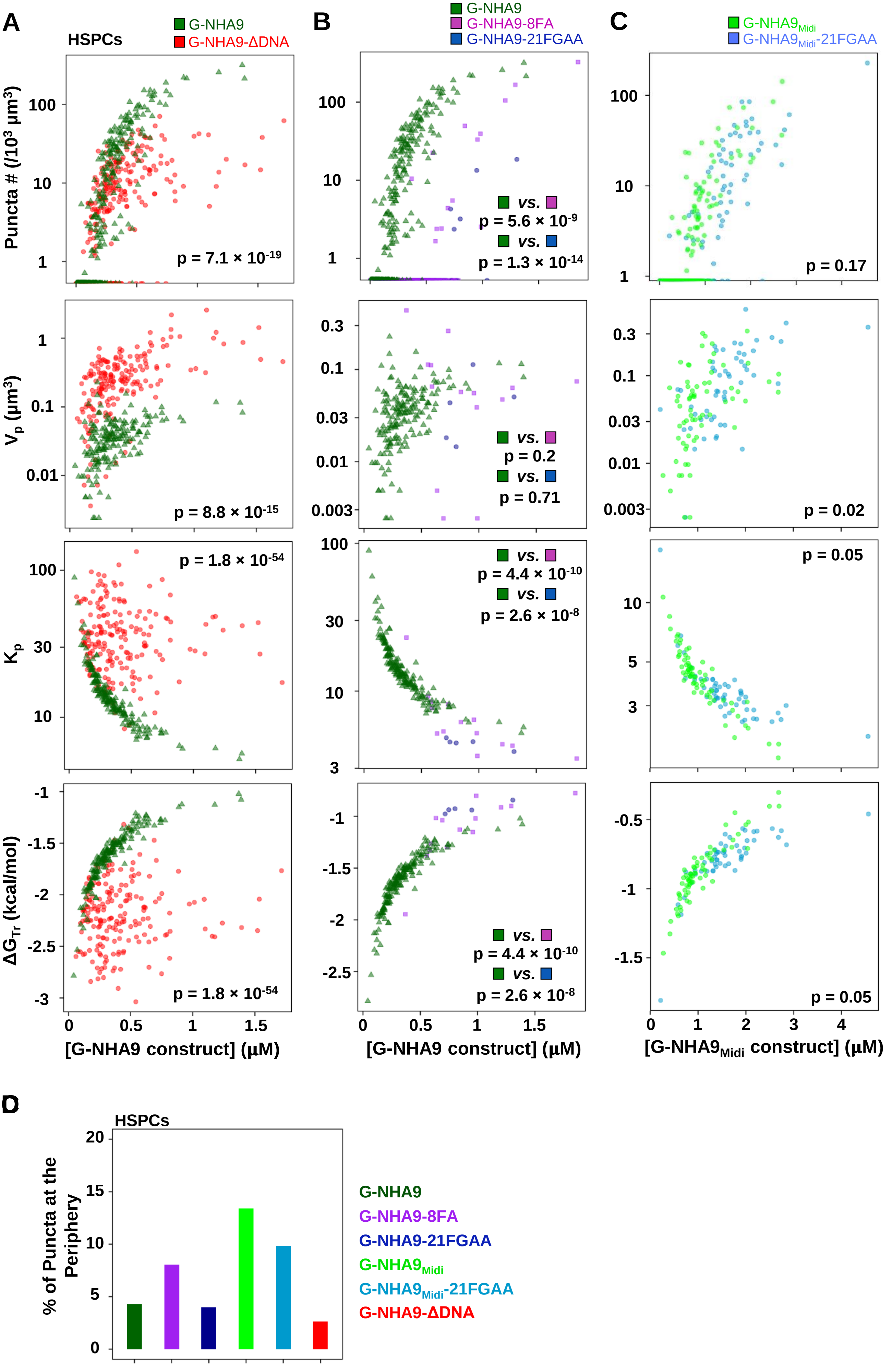

Suppl. Figure 5. Quantitative analysis of lin- HSPCs expressing NHA9 constructs. A-C, Plots of puncta # (/10^3 μm^3), V_p (μm^3), K_p ($K_p = \frac{[DP]}{[LP]}$), and ΔG_{Tr} (kcal/mol) *vs.* [G-NHA9 construct] for G-NHA9 (green) *vs* G-NHA9-ΔDNA (red) (**A**), G-NHA9 (green) *vs.* G-NHA9-8FA (purple) *vs.* G-NHA9-21FGAA (blue) (**B**), and G-$NHA9_{Midi}$ (lime green) *vs.* G-$NHA9_{Midi}$-21FGAA (light blue) (**C**). Data is plotted on a semi-log (y-axis: $\log_{10}$) scale except for the ΔG_{Tr} plots. The pair wise p-values between G-NHA9 *vs.* G-NHA9-ΔDNA, G-NHA9 *vs.* G-NHA9-8FA, G-NHA9 *vs.* G-NHA9-21FGAA, and G-$NHA9_{Midi}$ *vs.* G-$NHA9_{Midi}$-21FGAA are shown in each plot (**A-C**) (n = 416, 504, 616, 456, 674 and 407, respectively, for G-NHA9, G-NHA9-ΔDNA, G-NHA9-8FA, G-NHA9-21FGAA, G-$NHA9_{Midi}$ and G-$NHA9_{Midi}$-21FGAA in the puncta # plots; n = 180, 193, 14, 5, 67 and 54, respectively, for G-NHA9, G-NHA9-ΔDNA, G-NHA9-8FA, G-NHA9-21FGAA, G-$NHA9_{Midi}$ and G-$NHA9_{Midi}$-21FGAA in the V_p, K_p, and ΔG_{Tr} plots). **D,** Quantitation of percent of total puncta localized within 0.5 μm of the nuclear periphery for all G-NHA9 constructs in mHSPCs.

Suppl. Figure 6:

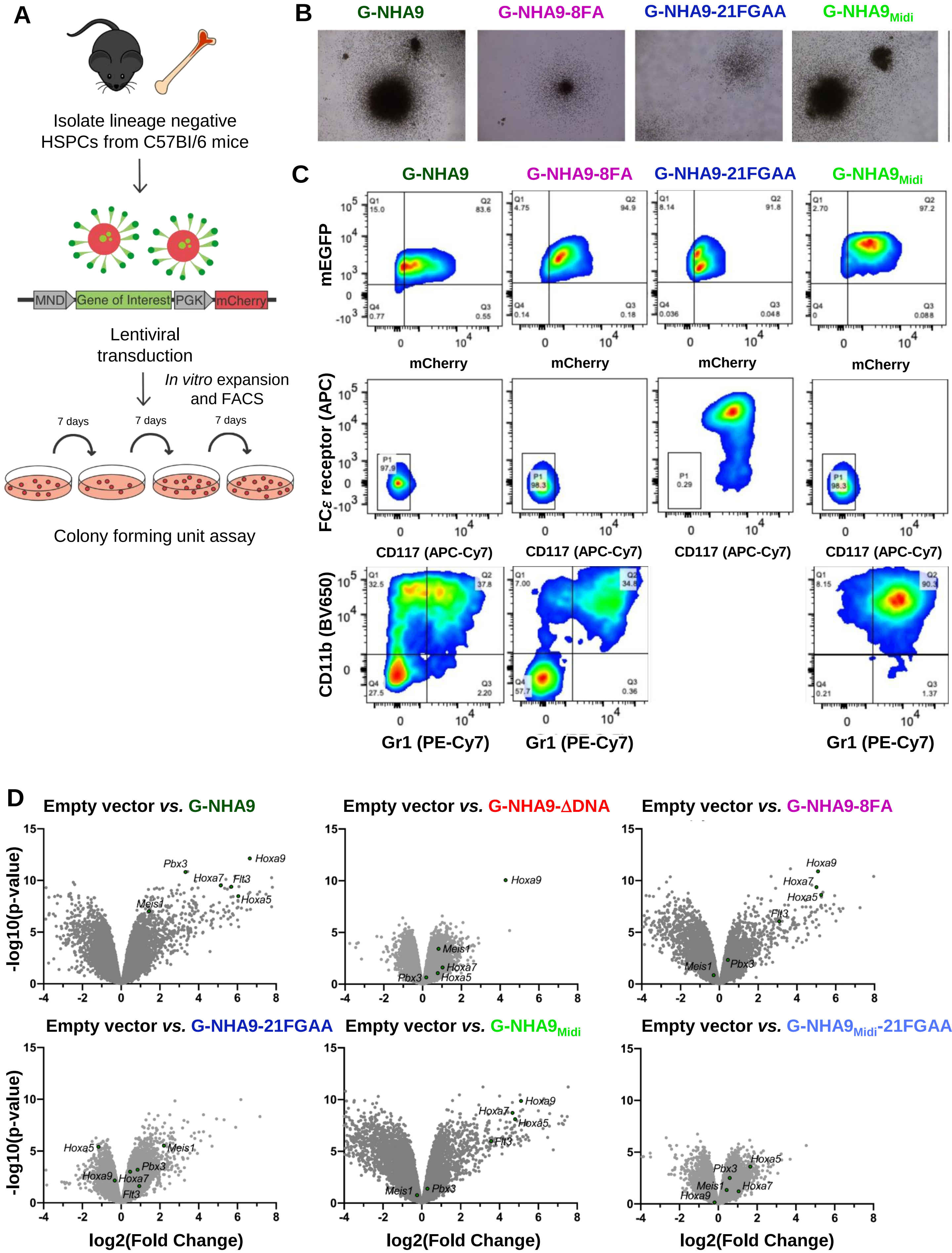

Suppl. Figure 6. Characterization of the developmental features of lin- HSPCs transduced with G-NHA9 constructs. A, Schematic for colony forming unit assay. **B**, Representative images of individual colonies from colony forming assays for lin- HSPCs expressing G-NHA9, G-NHA9-8FA, G-NHA9-21FGAA, and G-NHA9$_{Midi}$. **C**, Immunophenotyping of lin- HSPCs expressing G-NHA9, G-NHA9-8FA, G-NHA9-21FGAA, and G-NHA9$_{Midi}$ after three weeks of growth in methylcellulose containing myeloid and erythroid growth factors. Data shown are from the mCherry- and mEGFP-positive, CD117-negative, FCε receptor-negative live singlet population in a representative experiment. **D**, Volcano plots for RNA-seq data for empty vector *versus* G-NHA9 or mutants. RNA sequencing was performed for lin- HSPCs expressing empty vector, G-NHA9 or mutants after one week of growth in methylcellulose containing myeloid and erythroid growth factors. N = 5 for each condition.

Suppl. Figure 7:

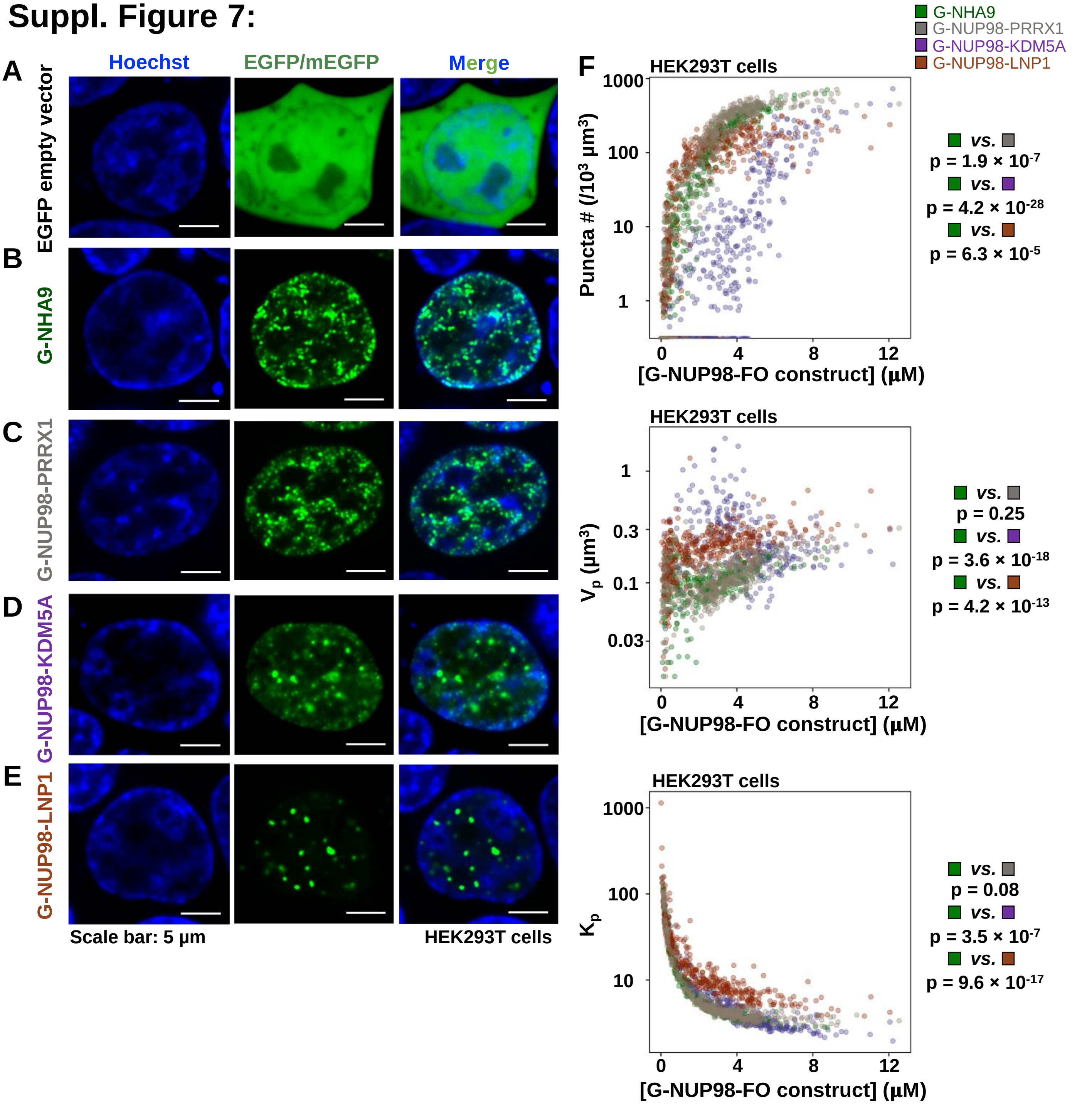

Suppl. Figure 7. Additional leukemia-associated NUP98 FOs form nuclear puncta in HEK293T cells and lin- HSPCs. A-E, Representative images of live HEK293T cells expressing EGFP empty vector (**A**), G-NHA9 (**B**), G-NUP98-PRRX1 (**C**), G-NUP98-KDM5A (**D**), and G-NUP98-LNP1 (**E**). DNA is stained with Hoechst dye (blue). **F,** Plots of puncta # (/10^3 μm^3), V_p (μm^3), and K_p ($K_p = \frac{[DP]}{[LP]}$) vs. [G-NUP98-FO construct] for G-NHA9 (green), G-NUP98-PRRX1 (grey), G-NUP98-KDM5A (purple), and G-NUP98-LNP1 (brown) from data represented in (**A-E**). Data is plotted on a semi-log (y-axis: $\log_{10}$) scale. The pairwise p-values between G-NHA9 *vs.* G-NUP98-PRRX1, G-NHA9 *vs.* G-NUP98-KDM5A, and G-NHA9 *vs.* G-NUP98-LNP1 are shown in each plot (**F**) (n = 972, 872, 998 and 1063, respectively, for G-NHA9, G-NUP98-PRRX1, G-NUP98-KDM5A and G-NUP98-LNP1 in puncta # plots including the cells with zero punctum; n = 352, 410, 277 and 373, respectively, for G-NHA9, G-NUP98-PRRX1, G-NUP98-KDM5A and G-NUP98-LNP1 in V_p and K_p plots excluding the cells with zero punctum).

Suppl. Figure 7 (Cont.):

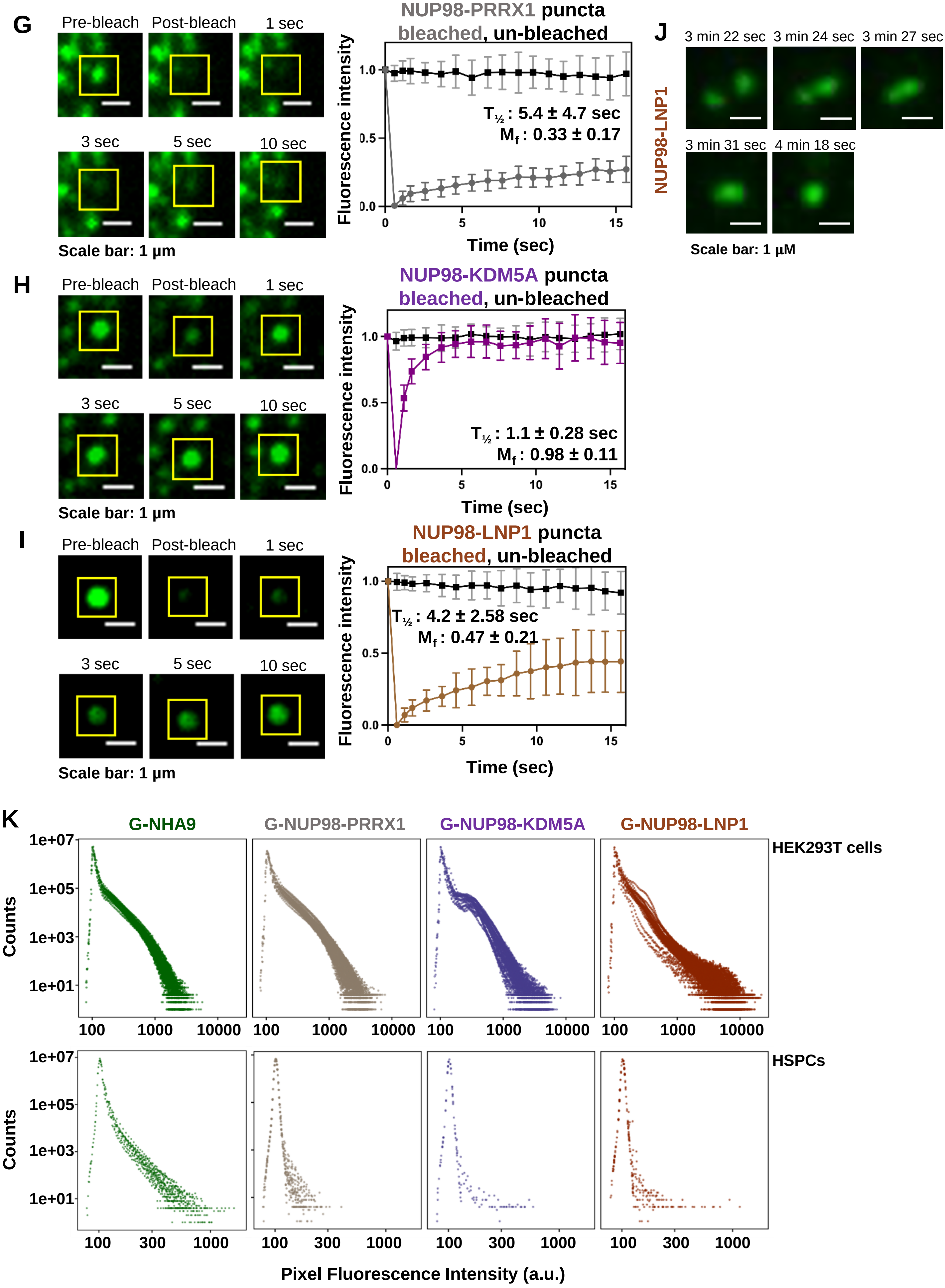

Suppl. Figure 7 (Cont.):

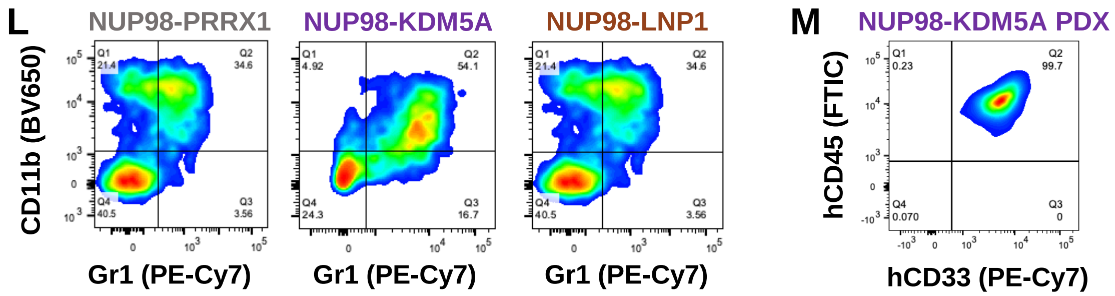

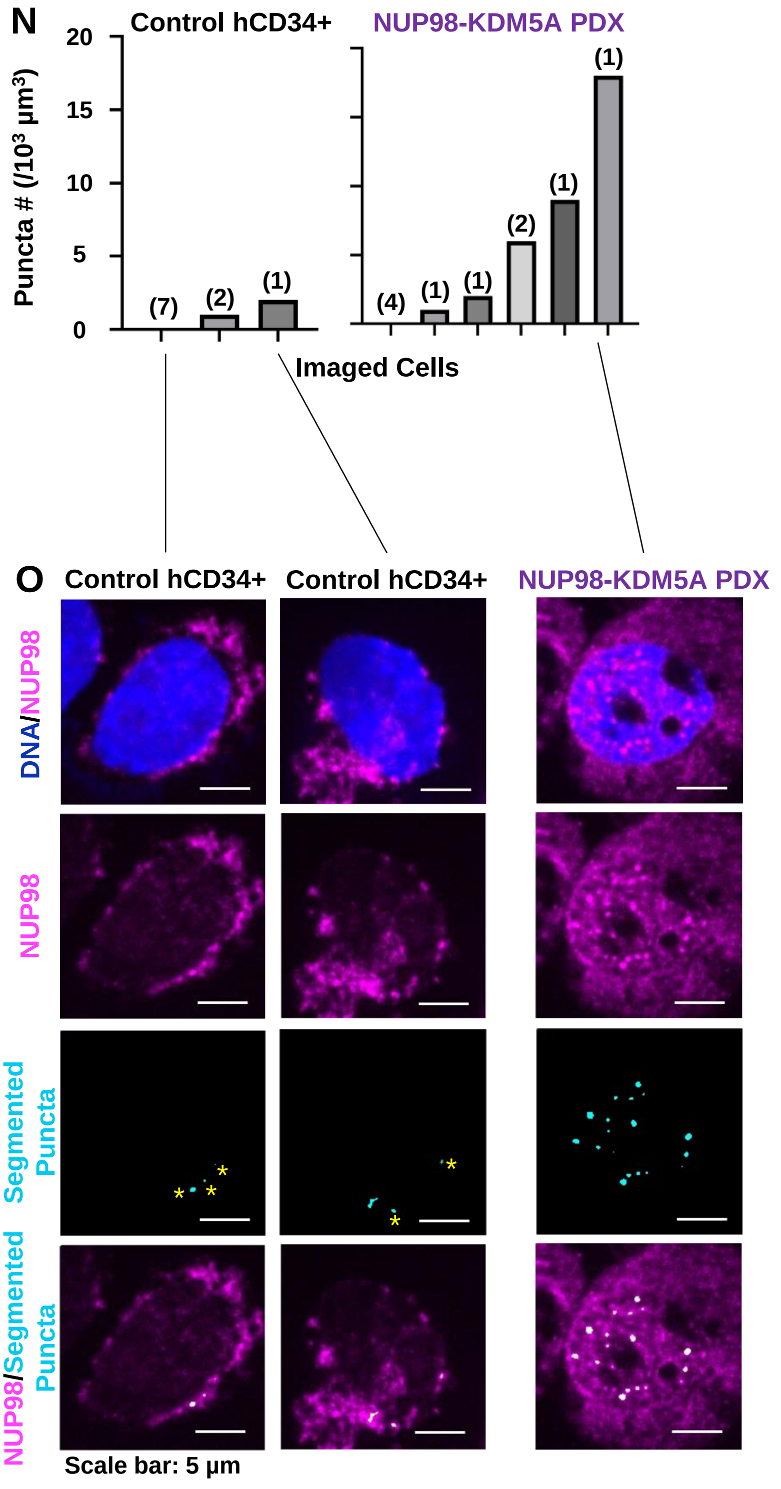

Suppl. Figure 7 (cont.). G-I, Confocal micrographs of fluorescence recovery (inside yellow box) of a single mEGFP-tagged punctum for G-NUP98-PRRX1 (**G**), G-NUP98-KDM5A (**H**), and G-NUP98-LNP1 (**I**) in HEK293T cells at different times after photo-bleaching (FRAP, left). FRAP recovery curves for the photo-bleached punctum are on the right. Individual puncta were manually tracked at different times and recovery was plotted as the mean ± the standard deviation (S.D.; n = 20). **J,** Still images of multiple time-points from a confocal fluorescence microscopy time-lapse video (**Suppl. Video 6**) for a fusion event in a HEK293T cell expressing G-NUP98-LNP1. **K,** 1D Histogram plots of mEGFP fluorescence intensity values extracted over all the pixels from the 3D images expressing NUP98-FOs in HEK293T (top) and mHSPCs (bottom). Data is plotted on a log-log (x-axis: $\log_{10}$ and y-axis: $\log_{10}$) scale. **L,** Immunophenotyping of lin- HSPCs expressing NUP98 FOs after two weeks of growth in methylcellulose containing myeloid and erythroid growth factors. Data shown are from the mEGFP-positive live singlet population in a representative experiment. **M,** Immunophenotyping of NUP98-KDM5A PDX cells extracted from a mass at tumor endpoint. Data shown are from the live singlet population. hCD45 and hCD33 refer to human CD45 and CD33, respectively. **N,** Quantification of segmented nuclear puncta in non-transduced human CD34+ cells (left) and NUP98-KDM5A PDX cells (right). Peripheral puncta are excluded (see Suppl. Methods). Numbers in parenthesis reflect the number of cells with the indicated number of puncta # (/10^3 μm^3), n = 10 cells for each condition. **O,** Representative image panels of data quantified in **(N)** with the indicated number of puncta. NUP98 is in magenta, DNA is in blue, and segmented puncta are in teal. Asterisks indicate peripheral NUP98 fluorescence intensity that was excluded in the quantification.

Suppl. Figure 8:

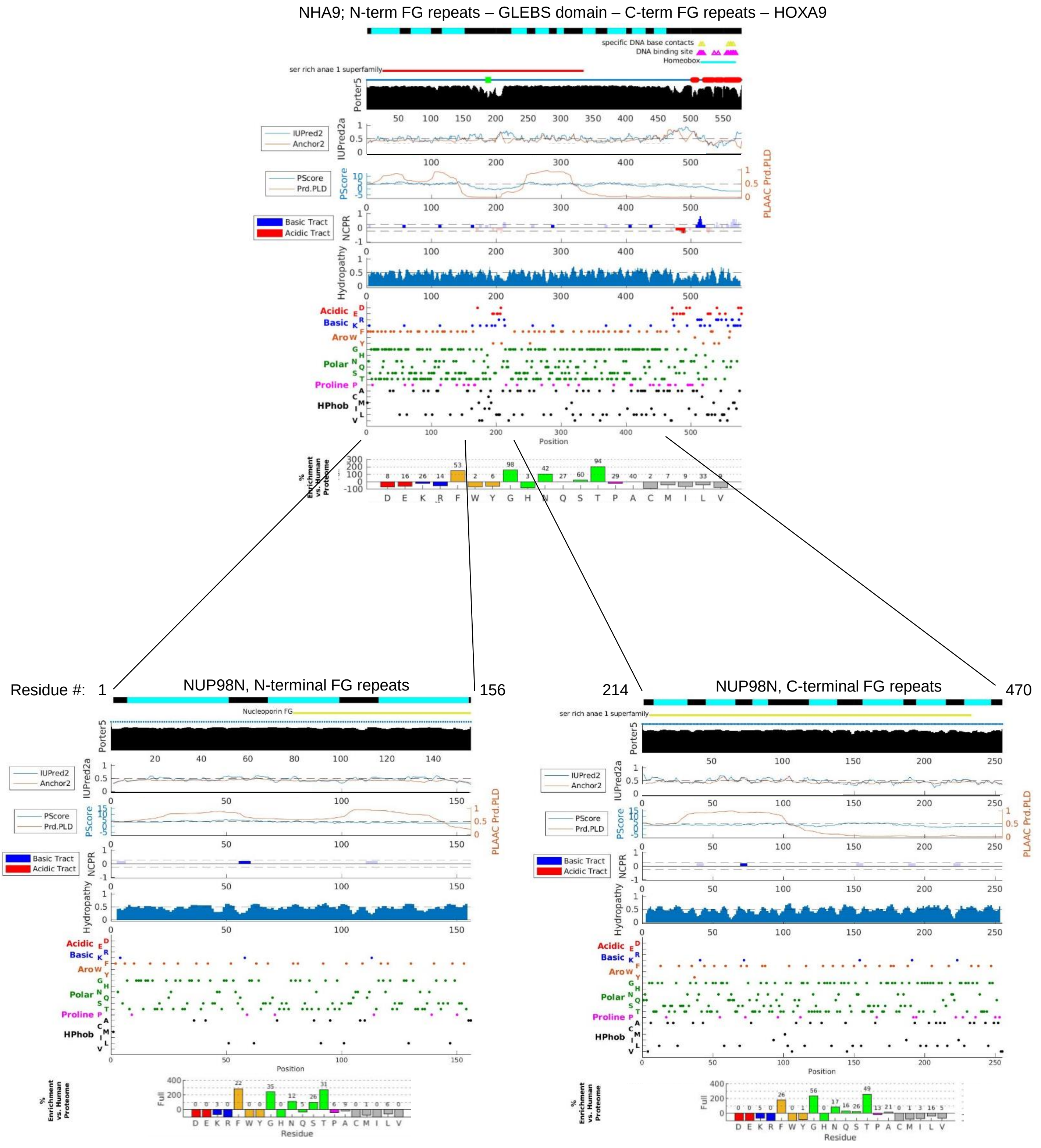

Suppl. Figure 8. Analysis of the amino acid features in the NHA9 sequence. Results of the sequence analysis pipeline show sequence features, including Shannon entropy, predicted secondary structure, predicted disorder, presence of cation-pi and pi-pi interactions, prion-like domains, acidic and basic tracts, and the occurrence and enrichment of amino acids within NHA9 (**top**), the N-terminal FG-motif region of NUP98-N within NHA9 (**bottom left**), and C-terminal FG-motif region of NUP98-N within NHA9 (**bottom right**). The number of residues in the regions are given above their respective bars in the bottom panel.
